# The GPI-anchor biosynthesis pathway is critical for syncytiotrophoblast differentiation and placental development

**DOI:** 10.1007/s00018-024-05284-2

**Published:** 2024-05-31

**Authors:** Andrea Álvarez-Sánchez, Johanna Grinat, Paula Doria-Borrell, Maravillas Mellado-López, Érica Pedrera-Alcócer, Marta Malenchini, Salvador Meseguer, Myriam Hemberger, Vicente Pérez-García

**Affiliations:** 1 Centro de Investigación Príncipe Felipe, Calle de Eduardo Primo Yúfera, 3, 46012 Valencia, Spain; 2 Epigenetics Programme, The Babraham Institute, Babraham Research Campus, Cambridge, UK; 3 Department of Biochemistry and Molecular Biology, Cumming School of Medicine, University of Calgary, Calgary, AB Canada; 4 Alberta Children’s Hospital Research Institute, University of Calgary, Calgary, Canada; 5 Centro de Biología Molecular Severo Ochoa, CSIC-UAM, Madrid, Spain

**Keywords:** Placental Syncytiotrophoblast, Trophoblast stem cells, CRISPR/Cas9 technology, Unfolded protein response, Endoplasmic reticulum stress, Preeclampsia

## Abstract

The glycosylphosphatidylinositol (GPI) biosynthetic pathway in the endoplasmic reticulum (ER) is crucial for generating GPI-anchored proteins (GPI-APs), which are translocated to the cell surface and play a vital role in cell signaling and adhesion. This study focuses on two integral components of the GPI pathway, the PIGL and PIGF proteins, and their significance in trophoblast biology. We show that GPI pathway mutations impact on placental development impairing the differentiation of the syncytiotrophoblast (SynT), and especially the SynT-II layer, which is essential for the establishment of the definitive nutrient exchange area within the placental labyrinth. CRISPR/Cas9 knockout of *Pigl* and *Pigf* in mouse trophoblast stem cells (mTSCs) confirms the role of these GPI enzymes in syncytiotrophoblast differentiation. Mechanistically, impaired GPI-AP generation induces an excessive unfolded protein response (UPR) in the ER in mTSCs growing in stem cell conditions, akin to what is observed in human preeclampsia. Upon differentiation, the impairment of the GPI pathway hinders the induction of WNT signaling for early SynT-II development. Remarkably, the transcriptomic profile of *Pigl*- and *Pigf*-deficient cells separates human patient placental samples into preeclampsia and control groups, suggesting an involvement of *Pigl* and *Pigf* in establishing a preeclamptic gene signature. Our study unveils the pivotal role of GPI biosynthesis in early placentation and uncovers a new preeclampsia gene expression profile associated with mutations in the GPI biosynthesis pathway, providing novel molecular insights into placental development with implications for enhanced patient stratification and timely interventions.

**Supplementary Information:**

The online version contains supplementary material available at 10.1007/s00018-024-05284-2.

## Introduction

The placenta is the essential, temporary extraembryonic organ that mediates the exchange of nutrients and oxygen between the mother and the embryo during pregnancy. The proper development of the embryo is reliant on a functional placenta, while abnormal placentation is linked to several pregnancy complications, including preeclampsia, fetal growth restriction, preterm birth, and in severe cases, miscarriage [1]. Even postnatally, placental insufficiency has long-lasting consequences on health and disease predisposition well into adulthood [2, 3]. Despite its critical role, the molecular mechanisms that regulate the development of the placenta remain incompletely understood.

Several systematic unbiased phenotypic screens in embryonic lethal mouse mutants have provided tremendous insight into gene functions and congenital disorders [4–7]. By examining the entire fetal-maternal unit, i.e. the embryo and the placenta, the “Deciphering the Molecular Mechanisms of Developmental Disorders” (DMDD) program showed that the prevalence of placental dysmorphologies in embryonic lethal mutants is far higher than previously appreciated. Moreover, phenotype co-association analysis revealed a significant correlation between placental abnormalities and, heart and brain development [8]. Further analysis of the DMDD database suggested that the glycosylphosphatidylinositol (GPI)-anchor biosynthesis pathway may play a pivotal role in regulating trophoblast differentiation and placenta development [9].

The GPI biosynthetic pathway is a highly conserved multistep process that culminates in the generation of the GPI glycolipid, which functions to anchor proteins to the cell surface. GPI-anchored proteins (GPI-APs) play diverse roles in eukaryotic organisms, serving as enzymes, adhesion molecules, complement regulators, and co-receptors in signal transduction pathways [10]. The GPI pathway involves more than 25 enzymes that catalyse the synthesis of GPI precursor molecules in the endoplasmic reticulum (ER) membrane. The GPI anchor is then post-translationally transferred to precursor proteins that have been translocated into the ER based on their N-terminal signal peptide. A unique C-terminal GPI signal sequence in these precursor proteins guides the attachment of the preassembled GPI anchor, before the mature GPI-AP is sent to the cell membrane via the secretory pathway [11]. Gene mutations or deletions in the components of the GPI biosynthesis system cause rare genetic disorders characterised by developmental delay, intellectual disability, seizures, dysmorphic features, and diverse congenital anomalies [12].

About one-third of these GPI pathway genes have been deleted in mouse models with most of these knockouts (7 out of 8) causing embryonic lethality [4]. We had previously reported that the deletion of three GPI pathway genes, *Pigl*, *Pigf,* and *Dpm1*, as well as a factor involved in the final modification phase, *Pgap2* (post-GPI attachment to proteins 2) results in embryonic lethality between embryonic day (E)9.5 and E14.5, a developmental time window associated with placental failure [8]. These data pointed to the GPI biosynthetic pathway as a potential new molecular hub in regulating early placentation.

**Fig. 1:**
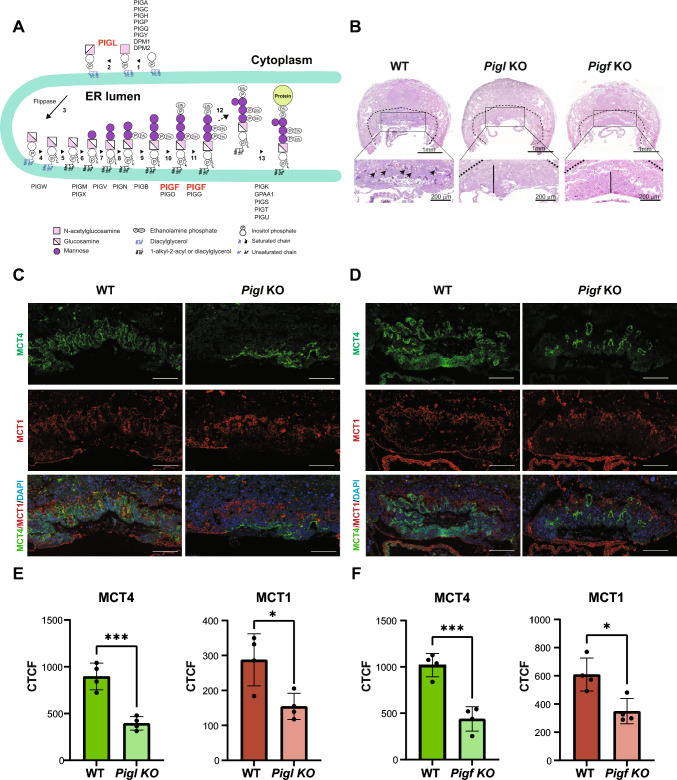
Analysis of placental defects in the *Pigl* and *Pigf* Knockout (KO) mice. **A** Schematic representation of the sequential assembly of the mammalian GPI in the endoplasmic reticulum (ER). The synthesis of the full GPI precursor capable of protein attachment occurs through consecutive enzymatic reactions (1)–(11), leading to the addition of the mannose-4 (Man4) side chain within the ER [step (12)]. The mature GPI assembly is then collectively transferred onto proteins in a single unit [step (13)]. Adapted from [10]. The associated genes corresponding to each reaction step are indicated beneath the respective step numbers. Highlighted in red are the main targets of this study, PIGL which catalyzes the initial step 2 of the pathway and PIGF involved in the regulation of final steps 10 and 11.** B** Representative hematoxylin and eosin staining of E9.5 placentas from a *Pigl* and *Pigf* KO, and littermate wild-type (WT) control. The dashed outline delineates the border between the maternal decidua and the trophoblast compartment. Scale bar = 1 mm. The insets zoom into the developing placental labyrinth zone. Scale bar = 200 µm. Black arrows (WT placenta): fetal blood vessels penetrating into the chorionic ectoderm. Vertical bars: unpatterned appearance of the chorion. Images are representative of ≥ 3 mutants per line. **C**, **D** Histological analysis of the placentas at E9.5 shows a strong placental defect in the syncytiotrophoblast layer development of *Pigl* KO mice (**C**) and *Pigf* KO mice (**D**). Sections were stained for MCT4 (SynT-II marker) and MCT1 (SynT-I marker). Nuclear counterstain with DAPI. Scale bar = 100 µm. Images are representative of at least 3 independent mutant placentas per line. **E**, **F** The bar plots show the mean quantification of MCT4 and MCT1 staining for *Pigl* KO (**E**) and *Pigf* KO (**F**) placentas compared to littermate WT controls, measured as CTCF (Corrected total cell fluorescence). Individual data points are shown as dots and error bars show standard deviation. Pairwise comparisons were done by a two-tailed t-test (*p < 0.05, ***p < 0.001)

Here we aimed to investigate the precise role of the GPI biosynthetic pathway in trophoblast biology. For this purpose, we focused on two ER-localized enzymes corresponding to the early and late steps in the GPI biosynthesis pathway: phosphatidylinositol glycan anchor biosynthesis class L protein (PIGL), which catalyzes the de-N-acetylation of GlcNAc-PI to GlcN-PI, the second step in GPI biosynthesis, and phosphatidylinositol glycan anchor biosynthesis class F protein (PIGF) which is involved in the ethanolamine phosphate (EtNP) transfer steps in late GPI biosynthesis [10] (Fig. 1A).

Detailed analysis of the placentation defects observed in the embryonic lethal mouse mutants for *Pigl* and *Pigf* reveals a severely underdeveloped labyrinthine syncytiotrophoblast (SynT), especially affecting layer SynT-II. By using CRISPR/Cas9 technology, we show that *Pigl-* and *Pigf-*deficient mouse trophoblast stem cells (mTSCs) fail to differentiate towards the syncytiotrophoblast lineage and instead preferentially differentiate into trophoblast giant cells. By functional and transcriptomic characterization of *Pigl-* and *Pigf*-deficient mTSCs grown in stem cell conditions and upon trophoblast differentiation, we show that impairment of the GPI biosynthesis results in suppression of pathways relevant for syncytiotrophoblast development such as WNT signaling, and causes an excessive activation of the unfolded protein response (UPR) in the ER. UPR is a common feature observed in human preeclampsia. A gene signature derived from *Pigl-* and *Pigf*-deficient mTSCs effectively separates human control and preeclampsia placental samples. Collectively, our data uncover an essential role for the GPI biosynthetic pathway in early placentation and reveal a novel signature in preeclamptic placentas that could be leveraged to enhance our understanding of the etiology of preeclampsia.

## Results

### Impaired placental labyrinth formation in *Pigl* and *Pigf* knockout mice

Previously, we had reported that the deletion of several components of the GPI biosynthesis pathway results in embryonic lethality at midgestation (E9.5–E14.5) and is associated with placental dysmorphologies affecting mostly placental labyrinth development [8]. For the particular case of *Pigl* and *Pigf* mutants, H&E analysis at E9.5 showed that they exhibited severe placental defects characterized by a lack of chorionic patterning and branching morphogenesis, and an absence of fetal blood vessels invading the chorion (Fig. 1B). To gain a more precise view of the structural defects in mutant placentas, we used immunofluorescence analysis for monocarboxylate transporters 1 (MCT1) and 4 (MCT4) which delineate the Syncytiotrophoblast (SynT)-I and SynT-II layers, respectively, that separate the maternal and fetal blood circulation. We observed a strongly underdeveloped SynT area in *Pigl-* and *Pigf-*deficient placentas, with the SynT-II layer being the most affected (Fig. 1C–F). Staining for E-cadherin (CDH1) and LAMININ to demarcate the labyrinthine syncytiotrophoblast and the basement membrane of fetal blood vessels, respectively, corroborated the mal-formation of the maternal–fetal interface in the placental labyrinth and revealed an impaired vascularisation in *Pigl* and *Pigf* mutant placentas (Fig. S1 A, B, C and D). The absence of *Pigl* induced a more severe placental phenotype in the labyrinth than was observed in *Pigf* deficient placentas, suggesting that these enzymatic reactions to generate GPI-APs are fundamental for the development of the labyrinthine syncytiotrophoblast (Fig. 1, and  Fig. S1). Overall, our data indicate that the defects in SynT development and vascularisation impaired fetal-maternal exchange in the *Pigl*^−/−^ and *Pigf*^−/−^ placentas, likely compromising fetal growth and survival.

### The absence of *Pigl* or *Pigf* does not affect stemness capacity in trophoblast stem cells

Mouse trophoblast stem cells represent the multipotent state of the extraembryonic trophoblast. They have the capacity to self-renew, and differentiate into the various trophoblast cell types that form the placenta when vital growth factors are withdrawn from the mTSC culture medium [13, 14]. In order to investigate the expression dynamics of the GPI pathway components *Pigl* and *Pigf* during trophoblast differentiation, we performed a 6-day mTSC differentiation time-course experiment. RT-qPCR analysis showed that, whereas *Pigl* expression did not significantly change across trophoblast differentiation (Fig. S2A), *Pigf* was highly expressed in mTSCs under stem cell conditions and was strongly downregulated during trophoblast differentiation (Fig. S2B). To study trophoblast-specific functions of these two GPI biosynthetic pathway enzymes, we generated *Pigl-* and *Pigf-*deficient mTSCs by using the CRISPR/Cas9 technology (Fig. S2C and D). When cultured in stem cell conditions, neither *Pigl* nor *Pigf* null mutants exhibited any significant changes in the protein levels of trophoblast stem cell markers such as CDX2 and ESRRB compared to controls (Fig. S2E and F). Moreover, no significant differences in cell morphology (Fig. S2G) or proliferation and migration rate were observed when the *Pigl*^*−/−*^ and *Pigf*^*−/−*^ mTSCs were subjected to wound healing assays in stem cell conditions (Fig. S2H). Altogether, our results indicate that the deletion of *Pigl* or *Pigf* does not impair the stem cell gene-regulatory network in mTSCs.

### Excessive UPR activation in the ER of *Pigl*^−/−^ and *Pigf*^−/−^ mTSCs

**Fig. 2:**
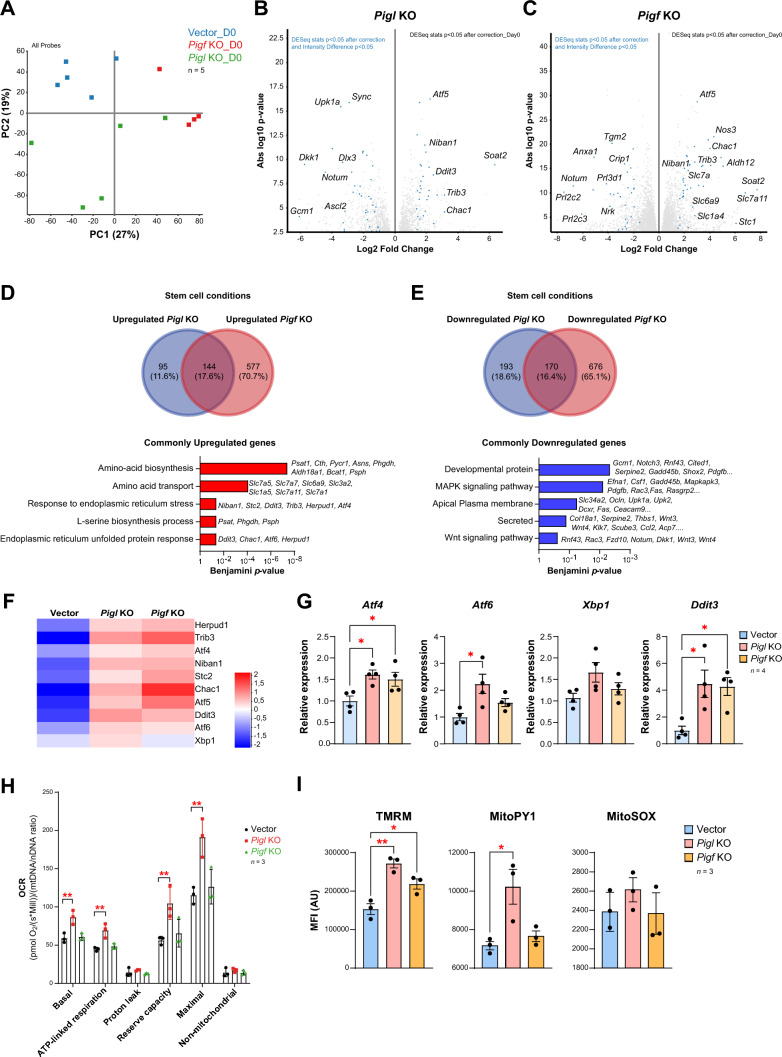
Ablation of *Pigl* and *Pigf* induces unfolded protein response (UPR) in mouse trophoblast stem cells. **A** Principal component analysis of global transcriptomes. **B** The volcano plot compares *Pigl* KO and vector control mTSCs for differentially expressed genes (DEGs) using DESeq2 and > 1-log2 fold change in expression. The most confident DEGs are highlighted in blue following DESeq2 and intensity difference analysis. **C** Equivalent analysis for *Pigf*-mutant mTSCs. **D**, **E** Venn diagram of genes commonly upregulated in *Pigl* KO and *Pigf* KO mTSCs (**D**), and conversely of genes commonly downregulated in *Pigl* KO and *Pigf* KO mTSCs (**E**), as identified by DESeq2 and > 1-log2 fold change in expression. Gene ontology analyses of genes in common are shown for each comparison. **F** Heatmap of mean row-centred log2 FPKM values of differentially expressed UPR genes (DESeq2 analysis) commonly dysregulated in *Pigl* KO and *Pigf* KO mTSCs. The heatmap shows a general UPR activation in the GPI pathway mutant mTSCs compared to vector control mTSCs. Five independent biological replicates per genotype were sequenced. **G** RT-qPCR analysis for the relevant UPR markers *Atf4*, *Atf6*, *Xbp1* and *Ddit3* (*Chop*) on vector control cells and *Pigl* KO and *Pigf* KO mTSCs growing in stem cell conditions. Data are mean ± SEM of *n* = 4 independent biological (cell clones) replicates. *p < 0.05 (one-way ANOVA with Dunnett’s multiple comparisons test). **H** Analysis of oxygen consumption rate (OCR) of vector control and *Pigl* or *Pigf* mutant mTSCs growing in stem cell conditions using different OXPHOS inhibitors. OCR was measured in each cell type under basal conditions and after sequential addition of oligomycin, carbonyl cyanide-p-trifluoromethoxyphenylhydrazone (CCCP), rotenone and antimycin A. The plot shows basal OCR (determined as the difference between OCR before oligomycin and OCR after rotenone/antimycin A), ATP-linked OCR (difference between OCR before and after oligomycin), proton leak (difference between basal OCR and ATP-linked OCR), reserve capacity (difference between the CCCP-stimulated rate and basal OCR), maximal OCR (difference between OCR after CCCP and non-mitochondrial OCR) and non-mitochondrial OCR (OCR after rotenone and antimycin A treatment). Data are mean ± SEM of *n* = 3 independent biological (cell clones) replicates. **p < 0.01 (one-way ANOVA with Dunnett’s multiple comparisons test).** I** Determination of mitochondrial membrane potential and Reactive Oxygen Species (ROS) by flow cytometry in vector control and *Pigl* KO and *Pigf* KO mTSCs with TMRM, and MitoPY1 and MitoSOX Red, respectively. Data are mean ± SEM of *n* = 3 independent biological (cell clones) replicates. *p < 0.05, **p < 0.01 (one-way ANOVA with Dunnett’s multiple comparisons test)

To determine the molecular impact of defective GPI anchor formation on the trophoblast, we studied the global expression profile of *Pigl*- and *Pigf*-mutant mTSCs compared to control cells grown in stem cell conditions. Principal component analysis (PCA) demonstrated that the differentially expressed genes (DEGs) were determined by the absence of *Pigl* or *Pigf* (Fig. 2A). By using DESeq2 and > 1-log2 fold change in expression, we identified 603 DEGs in *Pigl* knockout (240 upregulated, 363 downregulated) and 1568 in *Pigf* knockout mTSCs (722 upregulated, 847 downregulated) (Supplementary files 5 and 6). Gene ontology analysis on upregulated genes in *Pigl*^−/−^ mTSCs revealed an enrichment in terms related to UPR with deregulated genes such as *Atf4* and *Atf6*, *Ern1* (also known as *Ire1), Stc2* [15, 16], amino acid transmembrane transporters such as *Slc7a5* and *Slc7a7* [17], angiogenesis *(Vegfa)* [18], and mitochondrion (*Pycr1)* [19] (Fig. S2I and Supplementary file 7). Genes downregulated in *Pigl*^−/−^ mTSCs were associated with basal cell carcinoma, signaling pathways regulating pluripotency of stem cells, WNT signaling pathway genes such as *Wnt3* and *Wnt4* [20], and differentiation and MAPK signaling pathways, including essential genes involved in regulating placenta development such as *Gcm1*, *Cited1*, and *Ascl2* [9] (Fig. S2J and Supplementary file 7). Stringent calling of DEGs using DESeq2 and intensity difference analysis revealed that genes regulating WNT signaling such as *Dkk1, Dlx3* and *Notum* and trophoblast differentiation genes such as *Gcm1, Ascl2* were indeed strongly repressed, while several genes involved in the UPR in the ER such as *Niban1*, *Ddit3*, *Trib3* and *Chac1* [16] were upregulated in *Pigl* mutant mTSCs (Fig. 2B).

In *Pigf*^−/−^ mTSCs mutants, the DEG gene ontology analysis showed that upregulated genes were involved in the regulation of symport activity, amino-acid biosynthesis and transport, calcium signaling pathway and oxidation (Fig. S2K and Supplementary file 8). Downregulated genes in *Pigf*^−/−^ mTSCs were enriched in categories such as MHC class I peptide loading complex, glycoprotein, female pregnancy, cell adhesion molecules and MAPK signaling pathway (Fig. S2L and Supplementary file 8). Additionally, we found a cohort of 72 genes that were consistently deregulated (DESeq2 and Intensity difference analysis), out of which 40 genes were upregulated and 32 genes downregulated compared to vector control cells (Fig. 2C). Among the stringently upregulated genes in *Pigf*^−/−^ mTSCs were key regulators of symport activity, amino-acid biosynthesis and transport*,* and calcium signaling*.* The most repressed genes in *Pigf*^−/−^ mTSCs included glycoproteins like pregnancy-associated genes *Prl3d1*, *Prl2c* [21] and WNT signaling regulators such as *Notum, Crip1* and *Tgm2* [22–24].

To delineate the molecular mechanism by which the GPI biosynthesis pathway regulates the trophoblast compartment, we overlapped the transcriptome (DESeq2 and > 1-log2 Fold Change DEGs) of both *Pigl*- and *Pigf*-deficient mTSCs and determined the common deregulated genes. We found that 314 genes were commonly deregulated (144 upregulated and 170 downregulated genes) in both GPI pathway mutants (Fig. 2D and 2E). GO analysis of the commonly upregulated genes revealed involvement in amino acid biosynthesis, amino acid transport, response to ER stress and UPR (Fig. 2D and Supplementary file 9). The set of genes commonly downregulated in the GPI mutants included enrichment in categories and terms such as developmental protein, MAPK signaling pathway, apical plasma membrane, secreted proteins and WNT signaling pathway (Fig. 2E and Supplementary file 9).

Our transcriptomics data further suggest that the lack of *Pigl* and *Pigf* triggers UPR in the ER (Fig. 2F), which is likely due to the excess of misfolded proteins as a consequence of impaired GPI anchor formation [25]. To corroborate this finding, we assessed the expression of UPR marker genes by RT-qPCR and confirmed that *Pigl-* and *Pigf*-mutant mTSCs showed an upregulation of the key UPR markers *Atf4*, *Atf6*, *Xbp1*, and *Ddit3 (Chop)* compared to vector control cells (Fig. 2G), reflecting a general activation of the UPR in the absence of functional GPI biosynthesis pathway. In addition to the UPR, further adaptive cellular responses such as an increased mitochondrial respiration have been shown to be activated following ER stress in order to promote cell survival [26]. We analyzed mitochondrial respiration (the OXPHOS function) in *Pigl-* and *Pigf*-deficient mTSCs by measuring the oxygen consumption rate (OCR). *Pigl*-deficient mTSCs showed a significantly increased basal, ATP-linked, reserve respiratory capacity and maximal OCR compared to *Pigf* mutants and vector control cells (Fig. 2H). The ER stress and activation of mitochondrial respiration were accompanied by a significant increase in the mitochondrial membrane potential and Reactive Oxygen Species (ROS) production in the absence of *Pigl* and to some extent in the *Pigf*-deficient mTSCs compared to vector control mTSCs (Fig. 2I).

### The deletion of *Pigl* and *Pigf* impairs syncytiotrophoblast differentiation of mTSCs

**Fig. 3:**
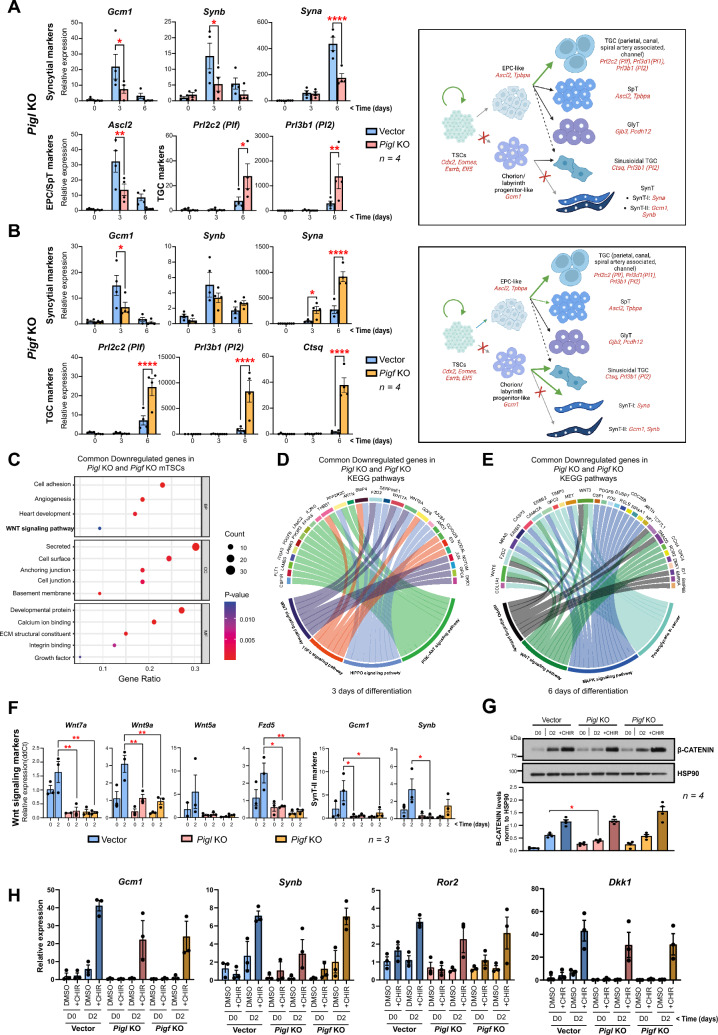
The deletion of *Pigl* and *Pigf* impairs syncytiotrophoblast differentiation of mTSCs. **A** Analysis of Vector and *Pigl* deficient mTSCs grown in self-renewal conditions (0 days) or after differentiation for 3 and 6 days assessed by RT-qPCR. Data are mean ± SEM of n = 4 independent biological (cell clones) replicates. *p < 0.05, **p < 0.01, ***p < 0.001, ****p < 0.0001 (two-way ANOVA with Sidak’s multiple comparisons test). **B** Equivalent analysis for *Pigf*^−/−^ mTSCs. The schematic diagrams **A**, **B** show the defects of differentiation observed in *Pigl*^−/−^ and *Pigf*^−/−^ mTSCs. EPC: ectoplacental cone; GlyT: glycogen cells; SpT: spongiotrophoblast; SynT: syncytiotrophoblast (layers I and II); TGC: trophoblast giant cells; TSCs: trophoblast stem cells. **C** GO enrichment analysis of the commonly downregulated genes in *Pigl* KO and *Pigf* KO mTSCs after 3 days of differentiation. **D** Chord plot indicates the relationship between genes and KEGG pathways in the commonly downregulated genes in *Pigl* KO and *Pigf* KO mTSCs after 3 days of differentiation. **E** Equivalent analysis for the commonly downregulated genes in *Pigl* KO and *Pigf* KO mTSCs after 6 days of differentiation.** F** RT-qPCR analysis for the WNT signaling pathway genes *Wnt7a*, *Wnt9a*, *Wnt5a*, *Fzd5* and the early syncytiotrophoblast (SynT-II) cell differentiation markers *Gcm1* and *Synb* on vector control cells and *Pigl* KO and *Pigf* KO mTSCs growing in stem cell conditions (0 days) and after 2 days of standard differentiation (2 days). Data are mean ± SEM of *n* = 3 independent biological (cell clones) replicates. *p < 0.05, **p < 0.01, (two-way ANOVA with Sidak’s multiple comparisons test).** G** Western blot analysis assessing the dynamic change in B-CATENIN levels on vector control cells and *Pigl* KO and *Pigf* KO mTSCs growing in stem cell conditions (D0) and after 2 days of standard differentiation (D2) or 2 days of differentiation in the presence of CHIR99021 (CHIR), which promotes differentiation specifically into syncytiotrophoblast layer-II cells (SynT-II). Quantification of band intensities of four independent biological (cell clones) replicate experiments is shown in the graph below. Data are normalized against HSP90 and are mean ± SEM; *p < 0.05 (two-way ANOVA with Sidak’s multiple comparisons test).** H** RT-qPCR analysis for the relevant syncytial differentiation markers *Gcm1*, *Synb, Ror2* and the WNT signaling target *Dkk1* on vector control cells and *Pigl* KO and *Pigf* KO mTSCs growing in stem cell conditions (D0) and after 2 days of standard differentiation (D2) or 2 days of differentiation in the presence of CHIR99021 (CHIR). Data are mean ± SEM of *n* = 3 independent biological (cell clones) replicates. (two-way ANOVA with Sidak’s multiple comparisons test)

To tease out the trophoblast-intrinsic defects induced by the lack of *Pigl* and *Pigf*, we subjected the *Pigl-* and *Pigf*-deficient mTSCs to a 6-day differentiation time-course experiment and analyzed trophoblast cell type-specific marker gene expression. When triggered to differentiate, *Pigl*^−/−^ and *Pigf*^*−/−*^ mTSCs failed to upregulate early markers of the SynT-II layer such as *Gcm1* and to a lesser extent *Synb,* indicating that differentiation towards the syncytiotrophoblast lineage was impaired in the absence of *Pigl* or *Pigf* (Fig. 3A and B, and Fig. S3A and B). The syncytiotrophoblast differentiation was more affected in *Pigl* mutants as they also showed lower expression of the SynT-I marker *Syna*, which was upregulated in the absence of *Pigf* (Fig. 3A and B). In addition, in both mutants the differentiation towards parietal and sinusoidal trophoblast giant cells (TGCs) was promoted (Fig. 3A and B, and Fig. S3 A and B). To further investigate the potential role of *Pigl* and *Pigf* in regulating TGCs, mTSCs were differentiated towards trophospheres in which mTSCs preferentially differentiate into TGCs [27, 28]. Whereas the morphology analysis did not reveal significant differences between control and mutant trophospheres, the upregulation of TGC markers such us *Tpbpa*, *Prl3d1*, *Prl3b1* and *Ctsq* in *Pigl-* and *Pigf-*deficient trophospheres confirmed a bias towards the differentiation of TGCs (Fig. S3C–E). Collectively, our findings indicate that the abnormal generation of the GPI in the ER of *Pigl* and *Pigf*-deficient mTSCs impairs the proper induction of the syncytial differentiation, confirming that the labyrinthine defects observed in the *Pigl* and *Pigf* KO mice are mainly due to a reduced capacity of the trophoblast compartment to develop the syncytiotrophoblast layer (Fig. 1, and Fig. S1A and B).

### GPI pathway defects impair the WNT signaling regulation of Syncytiotrophoblast-II cell differentiation

Given the decreased expression of several secreted and cell surface-associated regulators of WNT signaling upon lack of *Pigl* and *Pigf* in mTSCs grown in stem cell conditions (Fig. 2 and Supplementary file 9), we investigated whether the dysregulation of this essential pathway for syncytiotrophoblast differentiation [29, 30] could explain the labyrinthine defect observed in vivo (Fig. 1) and in vitro (Fig. 2). For this purpose, we analysed the global overlap in expression profiles of *Pigl*^−/−^ and *Pigf*^−/−^ mutant mTSCs compared to control cells at 3 days (3d) and 6 days (6d) of trophoblast differentiation by RNA-seq. Following 3d of differentiation, GO analysis of the commonly upregulated genes (DESeq2 analysis, 67 genes) showed an enrichment of genes associated with transcriptional regulation, negative regulation of ERK1 and ERK2 cascade, regulation of transcription from RNA polymerase II promoter, prolactin signaling and folate biosynthesis (Fig. S3F and G, Supplementary file 10). We found 184 genes commonly downregulated (DESeq2 analysis) in the absence of *Pigl* and *Pigf*, which showed strong enrichment in categories such as secreted proteins, cell surface molecules, cell adhesion, angiogenesis, and WNT signaling pathway (Fig. 3C and Supplementary file 10). KEGG pathway analysis revealed that several downregulated genes are involved in the regulation of the WNT, TGFβ, HIPPO and PI3K-AKT signaling pathways (Fig. 3D and Supplementary file 10). As differentiation progressed towards 6 days, the GO analysis of the commonly upregulated genes (DESeq2 analysis, 230 genes) included enrichment in categories such as negative regulation of cell proliferation, pregnancy, and trophoblast giant cell differentiation (Fig. S3 H and I, Supplementary file 10), corroborating the promotion towards TGC differentiation described above (Fig. 3A and B). Finally, the set of genes commonly downregulated in the GPI mutants after 6 days of differentiation (DESeq2 analysis, 240 genes) were enriched in regulators of WNT, HIPPO and MAPK signaling pathway (Fig. 3E, and Fig. S3 J, Supplementary file 10). Altogether, the transcriptomic data pointed towards the WNT signaling pathway as a molecular network that is consistently altered in the GPI mutant mTSCs and hence may underlie their defective differentiative capacity.

Interestingly, deregulated genes of the WNT signaling pathway at 3 days of differentiation largely comprised secreted and membrane-bound WNT components that have been described to be involved in triggering the early steps of SynT-II layer differentiation, such as *Wnt5a*, *Fzd5*, *Wnt7a* and *Wnt9a* (Fig. 3 and Fig. S3) [31–33]. This prompted us to assess the initial induction of the syncytial differentiation by performing a 2-day time-course experiment of trophoblast differentiation [8, 28]. *Pigl*^−/−^ and *Pigf*^−/−^ mutant mTSCs grown in stem cell conditions (0 days) showed a reduced expression of the WNT ligands *Wnt5a, Wnt7a, Wnt9a* and the WNT receptor *Fzd5* compared to control cells and failed to upregulate their expression upon differentiation, along with the early SynT-II markers *Gcm1* and *Synb* (Fig. 3F). To understand whether the decreased expression of WNT ligands and receptors impaired the WNT signaling activity in *Pigl*^−/−^ and *Pigf*^−/−^ mutant mTSCs, we assessed the level of B-CATENIN by Western blotting. The *Pigl*^−/−^ mTSCs and to a much lesser extent the *Pigf*^−/−^ mTSCs showed reduced B-CATENIN protein levels at 2 days of trophoblast differentiation, indicating an impairment of the WNT signaling pathway (Fig. 3G). Treatment of *Pigl-* and *Pigf*-deficient mTSCs with CHIR99021 (CHIR), which activates WNT signaling by stabilizing B-CATENIN and drives TSC differentiation specifically towards SynT-II cells [34], effectively upregulated B-CATENIN protein levels in both *Pigl* and *Pigf* knockout mTSCs (Fig. 3G), and induced the expression of the early SynT-II markers *Gcm1*, *Synb*, *Ror2* [35] and classical WNT target genes such as *Dkk1* to a level similar to control counterparts (Fig. 3H). These data indicate that the downregulation of secreted and cell surface-bound components of the WNT pathway in the GPI mutants impairs the early steps of the SynT-II cell differentiation and is a major contributor to the observed labyrinth phenotype.

### GPI pathway defects in mTSCs mirror a gene signature associated to human preeclampsia

**Fig. 4:**
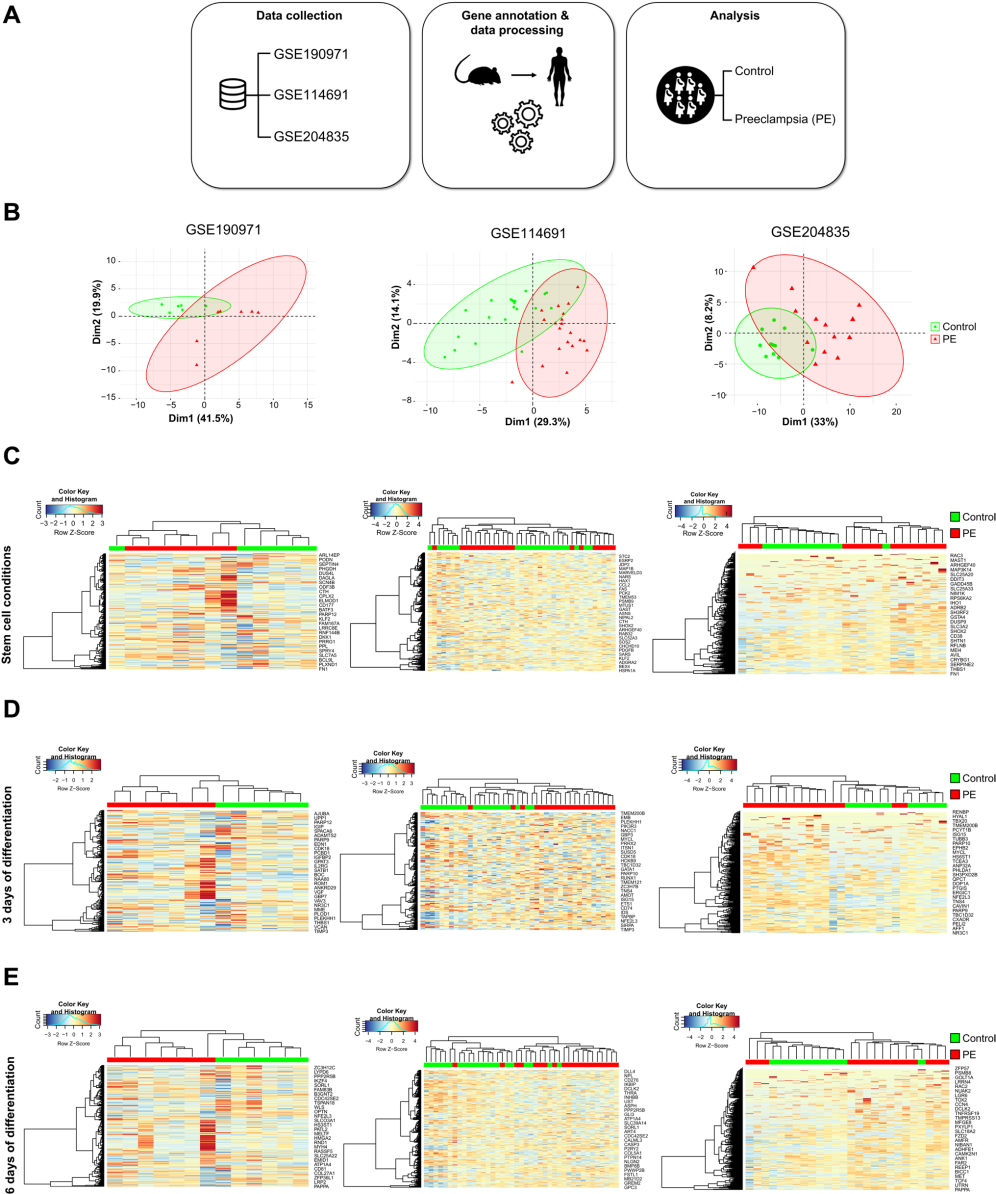
Validation of the GPI mutant gene signatures in human placental samples from preeclampsia datasets. **A** Schematic diagram of the study design. RNA-seq data from three different preeclampsia (PE) datasets (GSE190971, GSE114691, and GSE204835) were collected from the GEO database. Gene annotation and data processing including the normalization of RNA-seq counts matrix was applied in order to analyze the GPI gene signature. Principal component analysis (PCA) and unsupervised clustering validated the potential of the list of genes to separate the groups in each study.** B** PCA plots illustrating the distribution of PE (red) and control (green) samples from three datasets, based on the GPI gene signature of mTSCs grown in stem cell conditions. X and Y axis show dimension 1 and dimension 2 that explain the % of the total variance. **C** Unsupervised clustering based on the GPI gene signature of mTSCs grown in stem cell conditions across PE (red) and control (green) samples of the three datasets. **D** Equivalent analysis for the commonly deregulated genes in *Pigl* KO and *Pigf* KO mTSCs after 3 days of differentiation. **E** Equivalent analysis for the commonly deregulated genes in *Pigl* KO and *Pigf* KO mTSCs after 6 days of differentiation

It has been previously reported that placental UPR with impaired mitochondrial function is activated due to high levels of endoplasmic reticulum stress in preeclampsia [36, 37]. These observations prompted us to investigate whether the commonly deregulated genes of *Pigl-* or *Pigf-*mutant mTSCs grown in stem cell conditions and upon differentiation could represent signatures associated with GPI pathway defects and human preeclampsia. For this purpose, we interrogated our stem cell GPI mutant transcriptomic signature, defined as the 314 commonly dysregulated genes in both *Pigl-* and *Pigf*-deficient mTSCs (Fig. 3 and Supplementary file 9) against several public human preeclampsia (PE) data sets and other pregnancy disorders associated with a malfunctioning placenta such as intrauterine growth restriction (IUGR), gestational diabetes mellitus (GDM) or diseases where a potential link to placental dysmorphologies has been suggested such as COVID-19 (Fig. 4A and Fig. S4A, Supplementary file 11). Whereas PCA analysis based on our gene signature did not separate the data extracted from GDM, COVID-19 and IUGR, our signature gene set clustered PE and control samples separately into two groups in every preeclamptic dataset analyzed (Fig. 4B and Fig. S4B). Unsupervised hierarchical clustering analysis confirmed that the deficient GPI pathway signature effectively segregated the two groups: one encompassing mainly controls and the other mainly PE samples (Fig. 4C and Fig. S4C). These genes successfully grouped 81.6% of controls and 82.1% of PE cases, supporting this gene signature in PE [38]. The GPI gene signatures associated with differentiation, 251 genes and 472 genes commonly dysregulated in *Pigl*^−/−^ and *Pigf*^−/−^ mTSCs after 3 and 6 days of differentiation, respectively, were also able to separate PE placenta and control samples (Fig. 4D and E, and Fig. S4D and E). The gene signature corresponding to 3 days of differentiation grouped 87.4% of controls and 91.6% of PE samples and, the 6-day GPI gene signature discriminated 84.1% of controls and 91.1% of PE cases. Altogether, these results indicate that the gene signatures associated with an abnormal GPI biosynthesis pathway can represent a new identifying criterion of human preeclampsia.

## Discussion

The glycosylphosphatidylinositol (GPI) biosynthesis pathway is a key regulator of various cellular processes, including protein anchoring and signal transduction, with implications in development and disease [10, 12, 39]. We have previously reported that mouse embryos deficient for members of the GPI biosynthesis pathway, *Pigl*, *Pigf, Dpm1*, and *Pgap2*, exhibit lethality between E9.5 and E14.5 that is accompanied by profound placental abnormalities [8]. In the light of these data, we proposed the involvement of the GPI biosynthesis pathway in early placental development [9], but its molecular role in governing trophoblast development has remained unexplored thus far.

In the current study, we provide evidence that disruption of the GPI pathway, specifically through the knockout of *Pigl* and *Pigf* genes, perturbs syncytiotrophoblast differentiation and impairs placental labyrinth formation. The placental labyrinth exchange barrier is composed of three trophoblast cell types: a discontinuous layer of sinusoidal trophoblast giant cells, SynT-I and SynT-II cells – and an endothelial cell layer of the fetal vasculature. Many embryonically lethal mutations cause defects in the organization of the placental labyrinthine syncytiotrophoblast, which in turn compromises the development of the embryo [9, 40, 41].

Here we report a direct impact of *Pigl* and *Pigf* mutations on the development of the SynT-II layer. We show that the self-renewal capacity of *Pigl*- and *Pigf*-deficient mTSCs is preserved, but their differentiation into syncytiotrophoblast critically depends on a functional GPI biosynthesis pathway. Our transcriptomic data suggest two molecular mechanisms by which the GPI pathway disruption might impair syncytiotrophoblast differentiation. On the one hand, the *Pigl*- and *Pigf*-deficient mTSCs present with a strong UPR to endoplasmic reticulum stress. On the other hand, we observe repression of several components of the WNT signaling pathway during the early steps of syncytiotrophoblast differentiation in the absence of PIGL or PIGF.

Our transcriptomic data indicate that WNT signaling is one of the main pathways affected during trophoblast differentiation in the absence of PIGL and PIGF. The WNT pathway regulators which are repressed in *Pigf*^−/−^ and *Pigl*^−/−^ mTSCs and upon syncytiotrophoblast differentiation largely encode for secreted and membrane-tethered proteins (RNF43, NOTUM, DKK1, WNT3, WNT4, WNT5a, WNT7a, WNT9a, FZD5), which require glycosylation and palmitoylation in the ER [42]. Accordingly, their decreased expression might be explained by a negative feedback loop on their transcription, originating from a general defect in the secretory pathway associated with the abnormal generation of GPI-APs upon disruption of the GPI pathway. The lack of WNT ligands and their cell surface receptors in the GPI pathway mutants led to reduced levels of B-CATENIN protein, indicating reduced WNT signaling activity in the early steps of syncytiotrophoblast differentiation. It has been described that activation of the WNT signaling pathway through stabilization of B-CATENIN is essential for the expression of glial cell missing 1 (*Gcm1*) and its downstream target Syncytin b (*Synb*) to orchestrate SynT-II cell specification during placenta development [29, 30, 33, 43, 44]. Indeed, we observed that *Pigl*- and *Pigf*-deficient cells failed to upregulate *Gcm1* expression under differentiation conditions. As experimental activation of WNT signaling by CHIR treatment efficiently stabilized B-CATENIN protein levels and partially rescued the expression of *Gcm1* and *Synb* in *Pigl*- and *Pigf*-null mTSCs, we concluded that the downregulation of WNT pathway regulators is a major contributor to impaired syncytiotrophoblast differentiation in the GPI mutants.

Mutations in the GPI pathway can lead to an accumulation of misfolded GPI-APs in the ER, causing excessive ER stress. In response to ER stress, specific signal transduction events known as the UPR are activated, which aim to decrease the biosynthetic burden in the ER and restore protein folding homeostasis [45]. In addition to the UPR in the ER (UPR^ER^), further adaptive cellular responses such as an increased mitochondrial respiration have been shown to be activated following ER stress in order to increase antioxidant activity and promote cell survival [26]. The UPR^ER^ and mitochondrial UPR pathways (UPR^mt^) are closely linked, and activating one pathway is likely to trigger the other [46]. Our transcriptome data suggest that the impairment of the GPI biosynthesis pathway by either *Pigl* or *Pigf* deletion results in excessive ER stress, likely due to the accumulation of misfolded GPI-APs, triggering activation of the UPR via upregulation of *Atf4* and *Atf6* [47], and the mitochondrial UPR regulator *Atf5* [48] in *Pigl*- and *Pigf*-deficient mTSCs. We also detect increased mitochondrial respiration and, as a consequence, elevated mitochondrial ROS levels in the knockout mTSCs. Of note, in addition to its regulation by WNT signaling, expression of the syncytial marker *Gcm1* has been reported to be tightly regulated by UPR^ER^ and strongly decreased by elevated mitochondrial ROS levels in mouse and human trophoblast cells [49, 50], which can explain the impaired SynT-II differentiation of *Pigl*^−/−^ and *Pigf*^−/−^ mTSCs.

While a physiological level of UPR signaling has been associated with mammalian reproduction and placental function, among others through regulation of *Gcm1* [50], excessive ER stress and overactivation of UPR pathways, as observed in the *Pigl*- and *Pigf*-deficient mTSCs, have been associated with adverse pregnancy outcomes and placental dysmorphologies [51–54]. However, the precise mechanisms through which elevated ER stress influences early placental development have remained elusive. In a mouse model of chronic ER stress, a reduced volume of the labyrinthine zone has been reported, although without apparent effect on the structure of the syncytiotrophoblast [55]. In contrast, our data clearly show that both *Pigl* and *Pigf* mutants present an underdeveloped labyrinth caused by impaired syncytiotrophoblast differentiation. *Pigl*- and *Pigf*-deficient mTSCs are instead prone to differentiate into TGCs. In line with our results, a recent study has linked ER stress in mouse trophoblast stem cells to enhanced differentiation into Tpbpa + and Ctsq + TGC subtypes [52]. In-depth histological and functional assessments of the utero-placental architecture using transgenic mouse models manipulated to alter ER stress pathways will need to be conducted to precisely determine the impact of ER stress and UPR overactivation on trophoblast invasion, spiral artery remodeling, and the structural integrity of the labyrinth for nutrient exchange.

We have previously found a strong correlation between abnormal placentation and cardiovascular or brain defects in embryos [8, 9]. More recently, it has been reported that defects in mouse placental syncytiotrophoblast layers I and II formation are a common cause of developmental heart disease [56, 57]. Interestingly, human mutations affecting *PIGL* [58] and *PIGF* [59], and up to 15 other GPI biosynthesis pathway genes have been related to human genetic disease with common clinical features such as congenital heart defects and neurodevelopmental disorders [60]. The fact that deletion of the *Pigl* and *Pigf* genes in the mouse induces a severe SynT-II layer defect underscores the link between early placentation and heart and neural development in the fetus. A recent study has identified trophoblast-derived extracellular vesicles (EVs) as a potential communication pathway between the placenta and the nascent heart [61]. Differentiated trophoblast cells, especially syncytiotrophoblast cells, secrete abundant extracellular vesicles, which are detectable in both the maternal and fetal plasma [62]. Given that the formation of EVs is dependent on the ER [63] and that the deletion of *Pigl* and *Pigf* causes excessive ER stress, it is tempting to speculate that GPI pathway mutations might either reduce the secretion of syncytiotrophoblast extracellular vesicles (STB-EVs) by impairing syncytiotrophoblast differentiation or that the ER stress in *Pigl*- and *Pigf*-deficient placentas changes the cargo of STB-EVs [63, 64], ultimately depriving the fetus of cardio- and neuro-stimulating factors or promoting the transmission of pathological messages and thereby causing disease. In line with this thought, our transcriptome data of *Pigf*-null mTSCs reveal downregulation of *Anxa1*, a protein that has been associated with EV formation [65, 66], potentially indicating an impaired STB-EV biogenesis. With the *Pigl*^−/−^ and *Pigf*^−/−^ mTSCs at hand, it will be interesting to investigate the secretion and composition of STB-EVs secreted by the differentiating cells of these mutant mTSCs and to study their role in fetal heart and neural development.

One of the most striking implications of our study is the connection between GPI pathway defects and the pathogenesis of preeclampsia. Preeclampsia, a complex pregnancy-related disorder, is a major cause of maternal and fetal morbidity and mortality worldwide [67]. It has been previously reported that placental UPR with impaired mitochondrial function is activated due to high levels of persistent ER stress in the syncytiotrophoblast and drives the onset and persistence of preeclampsia [36, 37, 68]. On this background, we defined a gene signature linked to the *Pigl*- and *Pigf*-dependent alteration in the GPI biosynthesis pathway, which specifically identifies human preeclampsia samples and separates them from healthy controls. To the best of our knowledge, a direct link between *PIGL* or *PIGF* mutations and an increased risk of preeclampsia has not been reported so far. However, Deborde and colleagues [69] described a lack of GPI-PLD substrate in preeclamptic placentae and decreased serum levels of OMENTIN-1, a GPI-anchored protein, have been correlated with the presence and severity of preeclampsia [70]. In agreement with our findings, these studies suggest that a dysregulated GPI pathway plays a role in the pathophysiology of preeclampsia. The identification of a preeclamptic gene signature linked to defects in the GPI biosynthesis pathway offers a novel avenue for understanding the underpinnings of this enigmatic disorder. ER stress-induced UPR activation as well as a lack of secreted factors, driven by GPI pathway disruption, may contribute to the systemic inflammation, endothelial dysfunction, and oxidative stress characteristic of preeclampsia. This link between GPI pathway defects and preeclampsia pathogenesis highlights a novel area of investigation that may lead to the development of targeted therapeutic strategies and diagnostic biomarkers for this devastating disorder.

## Material and methods

### Animals

All mouse lines were produced and maintained on a C57BL/6N genetic background at the Wellcome Trust Sanger Institute (http://www.mousephenotype.org/) as part of the DMDD project [71]. Use of all animals was in accordance with UK Home Office regulations, the UK Animals (Scientific Procedures) Act of 1986, approved by the Wellcome Trust Sanger Institute’s Animal Welfare and Ethical Review Body and with approval of the local animal welfare committee (AWERB) at the Babraham Institute.

### Cell Culture and generation of mutant TSC lines

The wild-type TS-Rs26 TSC line (a kind gift of the Rossant lab, Toronto, Canada) and mutant TSC lines were grown as previously described [13]. Briefly, mTSCs were cultured in standard mTSC conditions: 20% fetal bovine serum (FBS) (Thermo Fisher Scientific 10270106), 1 mM sodium pyruvate (Thermo Fisher Scientific 11360-039), 1 × anti-mycotic/antibiotic (Thermo Fisher Scientific 15240-062), 50 μM 2-mercaptoethanol (Gibco 31350), 37.5 ng/ml bFGF (Cambridge Stem Cell Institute), and 1 μg/ml heparin in RPMI 1640 with L-glutamine (Thermo Fisher Scientific 21875-034), with 70% of the medium pre-conditioned on mouse embryonic fibroblasts (CM). The medium was changed every 2 days, and cells passaged before reaching confluency. Trypsinization (0.25% trypsin/EDTA) was carried out at 37 °C for about 5 min. The differentiation medium consisted of unconditioned TSC medium without bFGF and heparin. To induce SynT-II cell differentiation mTSCs were grown in TSC medium without bFGF2, heparin, and CM supplemented with 5 μM CHIR 99021 (Stemgent, 04-0004) following a modified protocol from [34]. The CHIR was solubilized in Dimethyl Sulfoxide (DMSO), which was used as a vehicle control during the experiments.

CRISPR/Cas9-mediated *Pigl*- and *Pigf*-Knockout (KO) mTSCs were generated as in [72]. Briefly, non-targeting gRNA (control) and gRNAs (Supplementary file 12) that result in frameshift mutations were designed using CRISPOR design software (http://crispor.tefor.net/) and cloned into the Cas9.2A.EGFP plasmid (Plasmid #48138 Addgene). Transfection of gRNA Cas9.2A.EGFP constructs was carried out with Lipofectamine 2000 (Thermo Fisher Scientific 11668019) reagent according to the manufacturer’s protocol. KO clones were confirmed by genotyping using primers spanning the deleted exon, and by RT-qPCR with primers within, and downstream of, the deleted exon, as shown (Fig. S2 C and D).

### Trophosphere generation

Trophospheres were generated following as in [27, 28]. In brief, 10^4^ wild-type and mutant cells resuspended in complete medium were cultured in Ultra-Low Attachment plates (Corning, Steuben County, NY). Forty-eight hours later, cells were collected, washed with PBS, and transferred back to Ultra-Low Attachment dishes with differentiation medium for another 7 days. Then, the trophospheres were collected for RNA analysis.

### Wound healing assay

Wound Healing Assay was performed to assess the ability of migration and proliferation between vector control cells and *Pigl* and *Pigf* deficient mTSC growing in stem cell conditions. Cells were seeded onto 96-well and the cell monolayer was scratched with a BioTek AutoScratchTM Wound Making Tool (Agilent) when they reached 95% confluence. Floating cells were removed with PBS, then the cells were incubated in fresh complete TSC for 24 h. Images were taken and quantified by BioTek CytationTM 5 Cell Imaging Multi-Mode Reader (BioTek, USA) equipped with its own cell BioSpa 8 incubator (BioTek, USA). The change in the wound area was calculated every 2 h and normalized to the baseline scratch area. The average migration speed was calculated by taking the mean of their individual speeds to the point the cells reached the plateaux in growth rate.

### Flow cytometry studies

After the indicated treatments, cells were detached at 37 °C with 0.25% trypsin/EDTA and resuspended in fresh TSC media. For ROS analysis, cells were incubated with 5 μM MitoSOX Red (Thermofisher, M36008) or MitoPY1 (Tocris, 4428/10) for 30 min at 37 °C, washed twice with phosphate buffered saline (PBS), and the red and the green fluorescence emitted respectively was measured. Mitochondrial activity based on membrane potential was assessed using TMRM (Thermofisher, T668) at a final concentration of 25 nm for 30 min at room temperature. For all the measurements, 10,000 cells were analysed and collected using a Cytomics FC 500 flow cytometer (Beckman Coulter).

### High resolution respirometry using oxygraph-2 K (Oroboros)

Oxygen consumption rate (OCR) in vector control cells and Pigl and Pigf deficient mTSCs was measured using a high-resolution respirometer (Oxygraph-2k, Oroboros Instruments, Innsbruck, Austria). Briefly, 80% confluent cells were detached at 37 °C with 0.25% trypsin/EDTA and resuspended in TSC medium at the concentration of 1000,000 cells/mL. Cells were simultaneously analyzed in two 2 mL-Oxygraph chambers. A real-time measurement of the OCR was performed at 37 C° in each chamber at basal conditions and after sequential addition of inhibitors for the different mitochondrial respiratory complexes: oligomycin (2.5 μM) to inhibit complex V (to assess non-mitochondrial respiratory capacity or leak rate), carbonyl cyanide-p-trifluoromethoxyphenylhydrazone (CCCP) uncoupler with stepwise titration in 0.5 μM increments (to assess maximal electron transport system respiratory capacity rate), rotenone (0.5 μM) to inhibit complex I, and antimycin A (2.5 μM) to inhibit complex III. Data was analyzed using DatLab7 (Oroboros, Austria) software. OCR values were normalized to the mitochondrial DNA copy number in each sample, quantified using RT-qPCR as described previously [73]. The specific primers utilized for this purpose were as follows: *CoxII*-Fw: CGATCCCTCCCTTACCATCA, *CoxII*-Rv: CCGTAGTCGGTGTACTCGTAGGT, *Sdha*-Fw: TCTCCAGTGGCCAACAGTGTT, *Sdha*-Rv: GCCCTCTTGTTCCCATCAAC.

### Western blot

Cells were lysed in radioimmunoprecipitation assay buffer (20 mM Tris–HCl, pH 8.0, 137 mM NaCl, 1 mM MgCl_2_, 1 mM CaCl_2_, 10% glycerol, 1% NP-40, 0.5% sodium deoxycholate, 0.1% sodium dodecyl sulphate), containing a protease inhibitor cocktail (Sigma P2714), and incubated at 4 °C for 1 h, followed by centrifugation (9300 × *g*, 10 min). Western blotting was performed as in [74]. Blots were probe against the antibodies anti-HSP90 (Cell Signaling, 4877), anti-b-CATENIN (D10A8) 1:1000 (Cell signaling, 8480T), anti-CDX2 1:1000 (Biogenex, MU392A-UC), anti-ESRRB 1:1000 (R&D Systems, H6707), followed by horseradish peroxidise-conjugated secondary antibodies anti-rabbit (Bio-Rad 170-6515), anti-mouse (Bio-Rad 170-6516, all 1:3000). Detection was carried out with enhanced chemiluminescence reaction (GE Healthcare RPN2209) on X-ray films. The intensity of the bands was quantified using ImageJ software.

### Immunofluorescence

For immunostaining, sections were deparaffinised in xylene and processed through an ethanol series to PBS. Antigen retrieval was performed by boiling in 1 mM EDTA pH 7.2, 0.05% Tween-20 followed by blocking in PBS, 0.5% BSA, 0.1% Tween-20. Antibodies used were anti-CDH1 (1:100; BD Biosciences 610,181), anti-LAMININ (1:100; Sigma L9393), anti-MCT1 (1:100; Merck Millipore AB1286I), anti-MCT4 (1:100; Merck Millipore AB3314P). Primary antibodies were detected with appropriate fluorescence or horseradish peroxidase-conjugated secondary antibodies; (1:400 Alexa594 (Goat anti-chicken) Invitrogen Cat. A110442, 1:400 Alexa488 (Donkey anti-rabbit) Invitrogen Cat. A21206, 1:200. Nuclei were counterstained with DAPI. For all immunostainings, samples were repeated at least twice in independent experiments. Images were taken with an Apotome fluorescence microscope (Apotome; Zeiss, Oberkochen, Germany) and quantified using ImageJ by calculating the corrected total cell fluorescence (CTCF), in arbitrary units, measured for each placental staining as follows: CTCF = integrated density—(selected area of the placental labyrinth × mean fluorescence of background readings). Before conducting the Student’s t-test to determine statistical significance, normality of each dataset was assessed using the Shapiro–Wilk normality test.

### RT-qPCR

Total RNA was extracted using TRI reagent (Sigma T9424), DNase-treated, and 1 µg used for cDNA synthesis with RevertAid H-Minus reverse transcriptase (Thermo Fisher Scientific EP0451). Quantitative (q)PCR was performed using SYBR Green Jump Start Taq Ready Mix (Sigma S4438) and Intron-spanning primer pairs (Supplementary file 12) on LightCycler 480 (Roche) according to the manufacturer’s specifications. Normalized expression levels are displayed as mean relative to the vector control sample; error bars indicate standard error of the means (SEM) of at least three replicates. Statistical testing for normality was performed for each data set by the Shapiro–Wilk normality test. Where appropriate, Student’s t-tests or ANOVA (one-way ANOVA with Dunnett’s multiple comparisons test or two-way ANOVA with Sidak’s multiple comparisons test) were performed to calculate the statistical significance of expression differences (p < 0.05) using GraphPad Prism 8.

### RNA-seq

For RNA-seq, total RNA was extracted with Trizol followed by DNase treatment using TURBO DNA-free kit (Life Technologies AM1907). RNA-seq libraries were generated from 500 ng using TruSeq Stranded mRNA library prep (Illumina, 20020594). Indexed libraries were pooled and sequenced on an Novaseq 6000 S4 sequencer in 150 bp paired-end mode. FastQ data were map to M. musculus GRCm38 genome assembly using HISAT2 v2.1.0. Sequence data were deposited in GEO under accession TBD.

### Bioinformatic analysis

Data were quantified using the RNA-seq quantitation pipeline in SeqMonk (http://www.bioinformatics.babraham.ac.uk) and normalized according to total read count (read per million mapped reads). Differential expression was calculated using DESeq2 and log2 Fold Change ≥ 1 with p < 0.05 and adjusted for multiple testing correction using the Benjamini–Hochberg method. Stringent differential expression was calculated combining DESeq2 and intensity difference filters in SeqMonk.

Heatmaps and PCA plots were generated using Seqmonk. Gene ontology was performed on genes found to be significantly up- or downregulated. Gene ontology terms with a Benjamini p-value of < 0.05 were found using DAVID [75]. Venn diagrams were plotted using Venny (https://bioinfogp.cnb.csic.es/tools/venny/).

For the assessment of the footprint defined in (Figs. 2, 3 and 4), bioinformatics analyses were performed using Preeclampsia (GSE190971, GSE114691, GSE204835), gestational diabetes mellitus (GDM) (GSE203346), COVID-19 (GSE171995), and intrauterine growth restriction (GSE220877) datasets from GEO database https://www.ncbi.nlm.nih.gov/geo/. Clinical data was downloaded from GEO database using GEOquery R package [76].

Data pre-processing included normalization of RNA-seq counts matrix with DESeq2 R package using variance stabilizing transformation (VST) method [75]. Gene annotation from Entrez and Ensembl identifiers to gene symbol was carried out with the AnnotationDbi R Package https://bioconductor.org/packages/AnnotationDbi. When dealing with duplicated genes, the median of their expression values was calculated.

Gene signature analysis included unsupervised clustering and Principal Component Analysis (PCA) to validate the potential of the list of genes to separate the different groups in each study. Unsupervised clustering at heatmaps was obtained by Euclidean measure with gplots (5) R package. Individual PCA plots were obtained with factoextra R package.

### Supplementary Information

Below is the link to the electronic supplementary material.

Supplementary file1 (PDF 622 KB)

Supplementary file2 (PDF 14727 KB)

Supplementary file3 (PDF 17964 KB)

Supplementary file4 (PDF 5491 KB)

Supplementary file5 (XLSX 108 KB)

Supplementary file6 (XLSX 267 KB)

Supplementary file7 (XLSX 103 KB)

Supplementary file8 (XLSX 229 KB)

Supplementary file9 (XLSX 80 KB)

Supplementary file10 (XLSX 198 KB)

Supplementary file11 (XLSX 104 KB)

Supplementary file12 (DOCX 27 KB)

Supplementary file13 (DOCX 3187 KB)

## Data Availability

All data generated or analysed during this study are included in the manuscript and supporting files. Genome-wide sequencing data have been deposited in the GEO database under accession number GSE266153.
