# Supplementary figures and images for: The GPI-anchor biosynthesis pathway is critical for syncytiotrophoblast differentiation and placental development
Source: Cell Mol Life Sci. 2024 May 31;81(1):246. doi: 10.1007/s00018-024-05284-2 (PMC11143174; doi:10.1007/s00018-024-05284-2)

**A**

WT

*Pigl* KO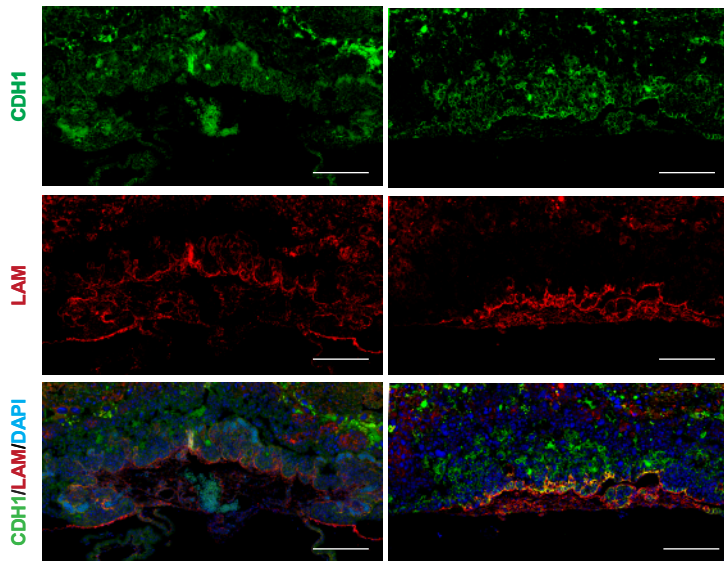**B**

WT

*Pigf* KO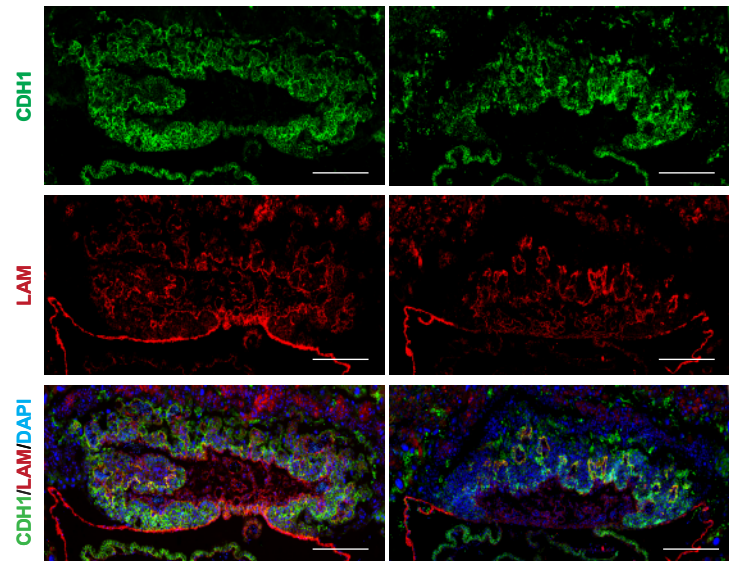**C**

CDH1

LAM

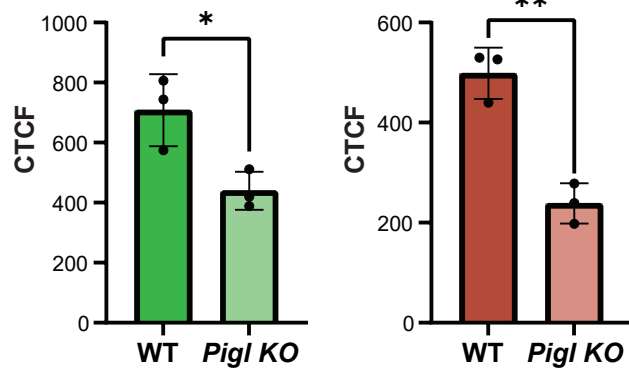**D**

CDH1

LAM

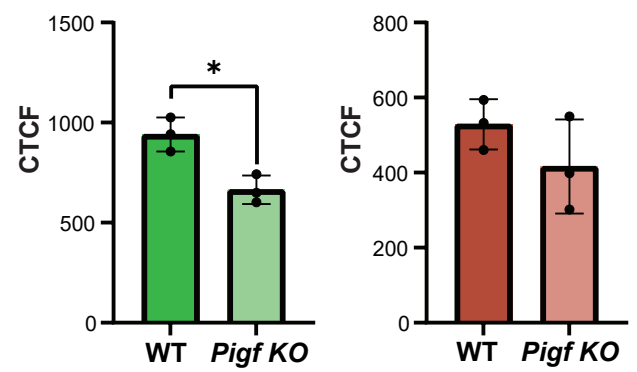

Supplement: Supplementary file 1 — Supplementary file1 (PDF 622 KB) [file 18_2024_5284_MOESM1_ESM.pdf]

A

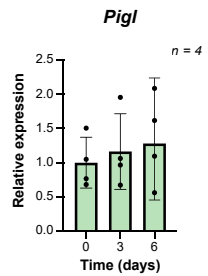

B

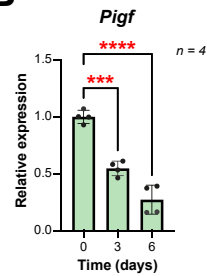

C

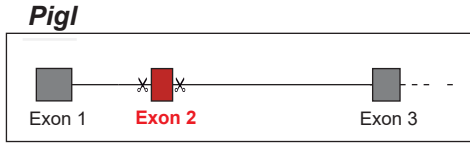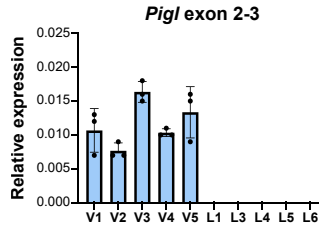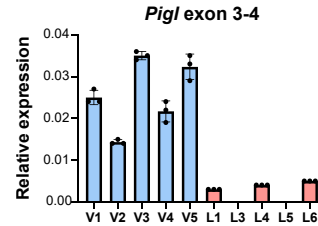

D

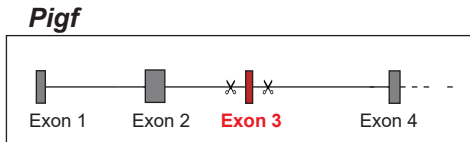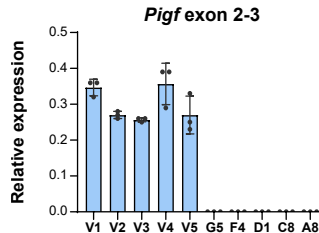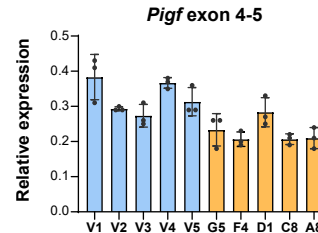

E

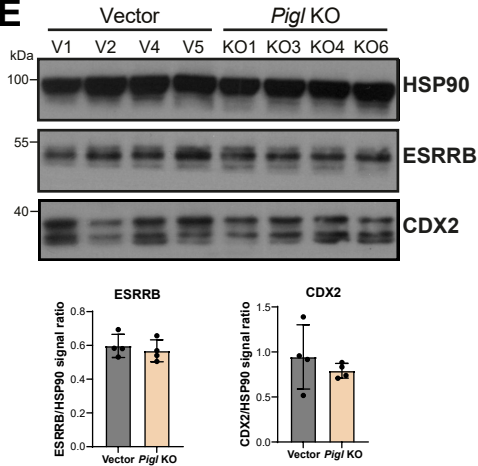

F

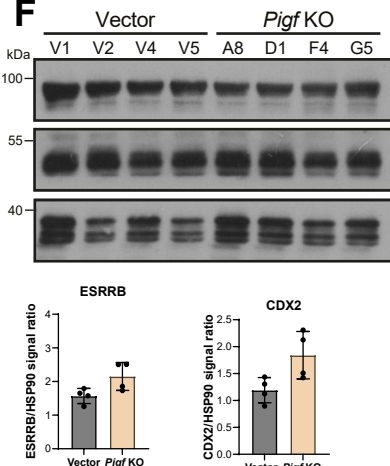

G

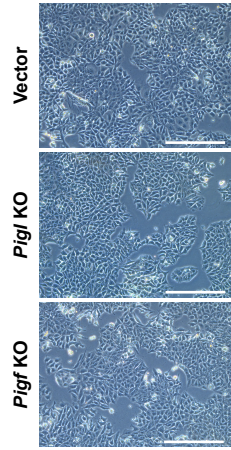

H

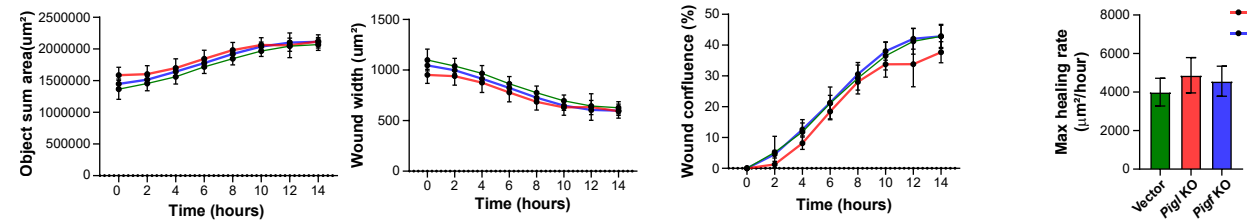

I

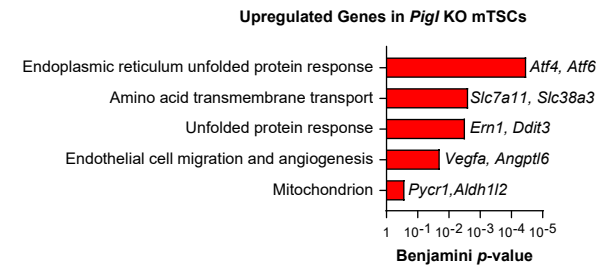

J

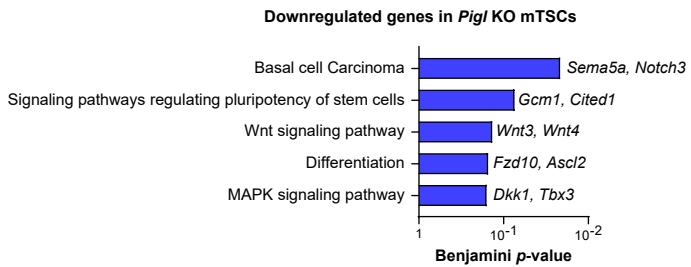

K

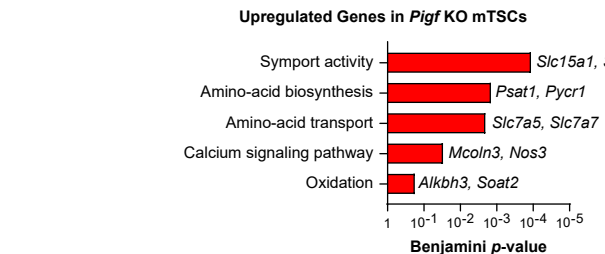

L

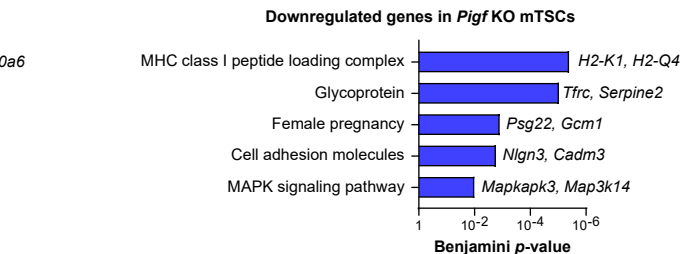

Supplement: Supplementary file 2 — Supplementary file2 (PDF 14727 KB) [file 18_2024_5284_MOESM2_ESM.pdf]

# Supplementary Figure 3

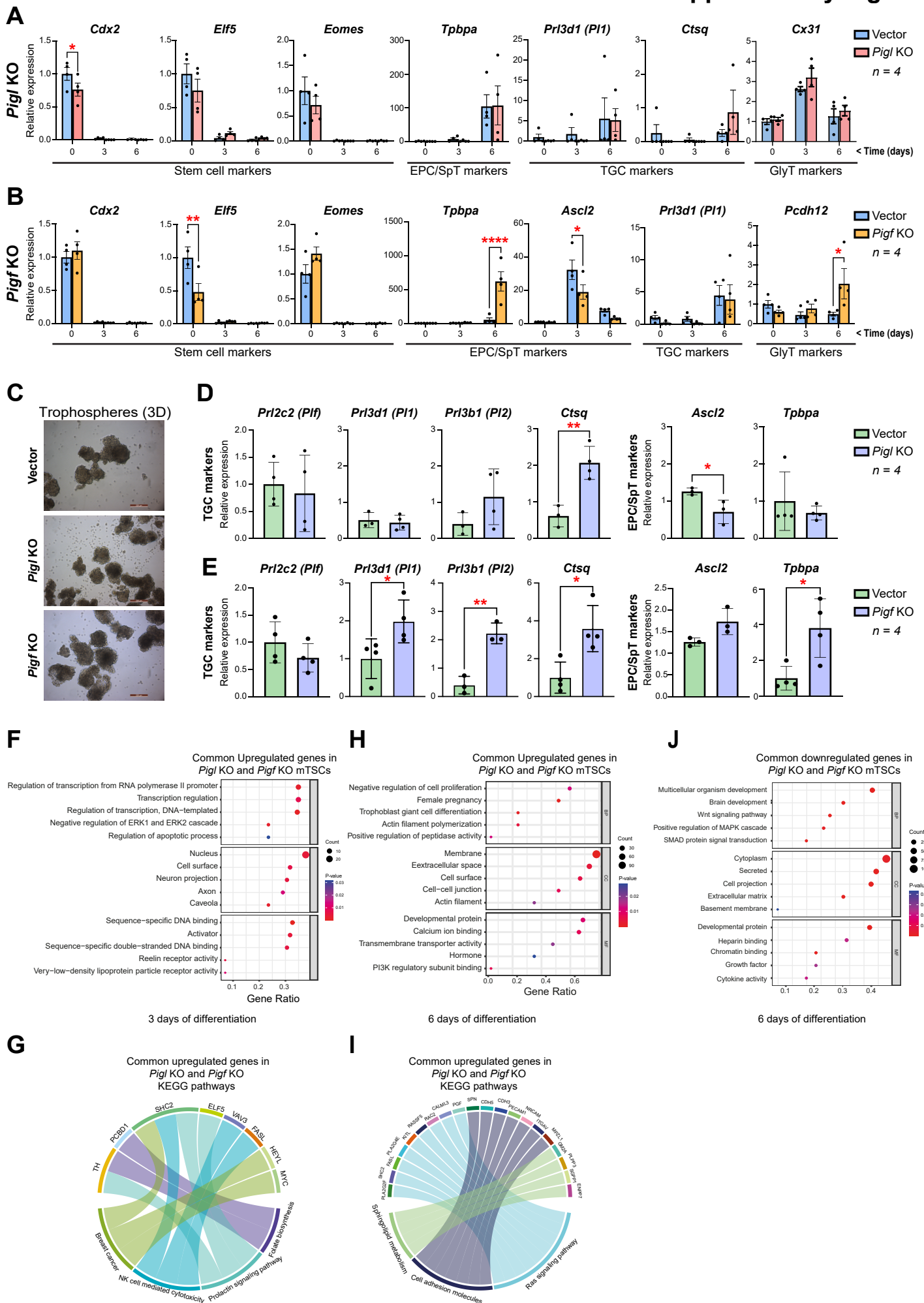

Supplement: Supplementary file 3 — Supplementary file3 (PDF 17964 KB) [file 18_2024_5284_MOESM3_ESM.pdf]

**A**

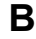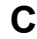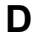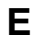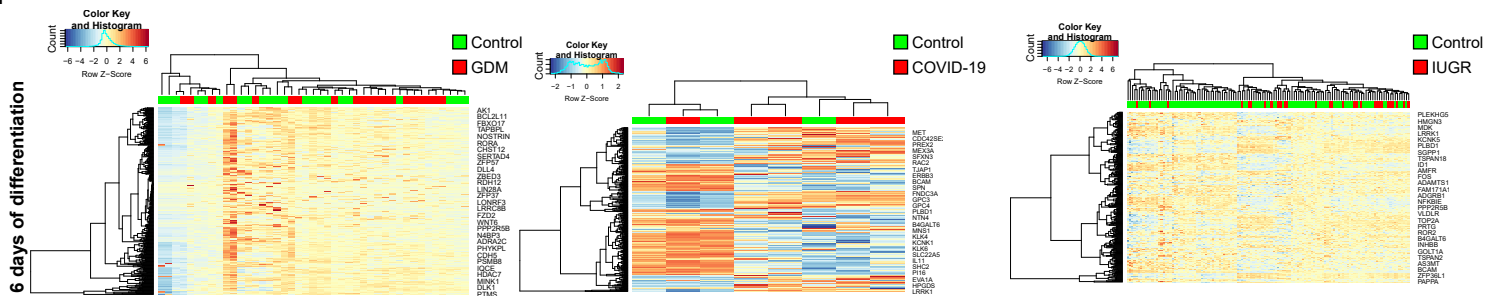

Supplement: Supplementary file 4 — Supplementary file4 (PDF 5491 KB) [file 18_2024_5284_MOESM4_ESM.pdf]
