# Supplementary material for: The GPI-anchor biosynthesis pathway is critical for syncytiotrophoblast differentiation and placental development
Source: Cell Mol Life Sci. 2024 May 31;81(1):246. doi: 10.1007/s00018-024-05284-2 (PMC11143174; doi:10.1007/s00018-024-05284-2)
Supplement: Supplementary file 12 — Supplementary file12 (DOCX 27 KB) [file 18_2024_5284_MOESM12_ESM.docx]

**Supplementary file 12: Primer sequences for RT-qPCR and CRISPR gRNAs target sequences**

| **Gene** | **Sequence** | |
| --- | --- | --- |
| *Ascl2* | F | AGCCCGATGGAGCAGGAG |
|  | R | CCGAGCAGAGGTCAGTCAGC |
| *Atf4* | F | GAGCTTCCTGAACAGCGAAGTG |
|  | R | TGGCCACCTCCAGATAGTCATC |
| *Atf6* | F | TCGCCTTTTAGTCCGGTTCTT |
|  | R | GGCTCCATAGGTCTGACTCC |
| *Cdx2* | F | AGTGAGCTGGCTGCCACACT |
|  | R | GCTGCTGCTGCTTCTTCTTGA |
| *Chop* | F | CTGCCTTTCACCTTGGAGAC |
|  | R | CGTTTCCTGGGGATGAGATA |
| *Ctsq* | F | AATTGGCTATGGTTATGTGGGA |
|  | R | TCACACAGTAGGGTATTGGG |
| *Dkk1* | F | CTCTTGACAACTACCAGCCCT |
|  | R | AGCACATAGCGTGCCTCAT |
| *Elf5* | F | ATTCGCTCGCAAGGTTACTCC |
|  | R | GGATGCCACAGTTCTCTTCAGG |
| *Eomes* | F | TCGCTGTGACGGCCTACCAA |
|  | R | AGGGGAATCCGTGGGAGATGGA |
| *Esrrb* | F | AGTACAAGCGACGGCTGG |
|  | R | CCTAGTAGATTCGAGACGATCTTAGTCA |
| *Fzd5* | F | TGTCGTTAAACTTTCCCAGCTCT |
|  | R | CTCCAAGGACAGAACTCTCGGA |
| *Gcm1* | F | ACCCCTGAAGCTTATTCCCT |
|  | R | TCGCCTTTGGACTGGAAA |
| *Pcdh12* | F | GCTGCTTTTGCGGAACGGAA |
|  | R | TTTGGGCTGGAATTGGCCCT |
| *Pigf (exon 2-3)* | F | TCTGCTCTGCTTCTGTGACCA |
|  | R | CAGCATTTCAAAGCTCGCGT |
| *Pigf (exon 4-5)* | F | ACCAAACCTCAAAGCATGGCTG |
|  | R | AGCCACGCTCCTGTGAAACT |
| *Pigl (exon 2-3)* | F | GCTGTGCTGTGTTGGGGATT |
|  | R | TGTCCCACTGCACTTCTGGA |
| *Pigl (exon 3-4)* | F | AGCACCCTCCTTCAGCACATAC |
|  | R | GCTGTGACCACTGACTCCTTC |
| *Prl2c2 (Plf)* | F | AACGCAGTCCGGAACGGGG |
|  | R | TGTCTAGGCAGCTGATCATGCCA |
| *Prl3b1 (Pl2)* | F | GCACTCGGGGAACAGCAGCC |
|  | R | ACTGCCAGCAACAGGAGTGCC |
| *Prl3d1 (Pl1)* | F | TTATCTTGGCCGCAGATGTGT |
|  | R | GGAGTATGGATGGAAGCAGTATGAC |
| *Ror2* | F | CTTCCCACTCTGAAAGGCTACT |
|  | R | CTTCGTGGCTCTTGCACAAC |
| *Rpl19* | F | CGAATGCCAGAGAAGGTCAC |
|  | R | CCATGAGAATCCGCTTGTTT |
| *Sdha* | F | TGGTGAGAACAAGAAGGCATCA |
|  | R | CGCCTACAACCACAGCATCA |
| *Syna* | F | CCTCACCTCCCAGGCCCCTC |
|  | R | GGCAGGGAGTTTGCCCACGA |
| *Synb* | F | TCCGGAAAGGGACCTGCCCA |
|  | R | CAGCAGTAGTGCGGGGTGCC |
| *Tpbpa* | F | ACTGGAGTGCCCAGCACAGC |
|  | R | GCAGTTCAGCATCCAACTGCG |
| *Wnt5a* | F | GCTTTGGATTGTCCCCCAAG |
|  | R | ATTCCAATGGGCTTCTTCATGG |
| *Wnt7a* | F | GAGATCAAGCAGAATGCCCG |
|  | R | TTCTCCTCCAGGATCTTCCGA |
| *Wnt9a* | F | CCTCGTGGGTGTGAAGGTGATA |
|  | R | CTTCATTGGTAGTGCTGCCC |
| *Xbp1* | F | G AGTCCGCAGCAGGTG |
|  | R | GTGTCAGAGTCCATGGGA |

**PCR screening primers**

| *Pigf* | F | ACAAAACTGGGTAAATTGCATCA |
| --- | --- | --- |
|  | R | AGCTAACAGCTAAAACCTATGA |
| *Pigl* | F | GATGCATAGGCATTGTAAATGGTGG |
|  | R | CCCTGCCAAGAAAAGCCAAG |

**CRISPR gRNAs target sequences**

| Pigl (exon 2) | Upstream | TCAGACGCTCTAAATCCCCC |
| --- | --- | --- |
|  |  | GGGGATTTAGAGCGTCTGAA |
|  | Downstream | GCTGTAGGTACTAACTAGCC |
|  |  | CTACAGCATGTGCGAAGTCC |
| *Pigf (exon 3)* | Upstream | GAACGTTCAGATCACGTTCT |
|  |  | TCAGATCACGTTCTAGGCAT |
|  | Downstream | TCCATTAACCAACCCCACCG |
|  |  | GCCACGGTGGGGTTGGTTAA |
