# Supplementary material for: The GPI-anchor biosynthesis pathway is critical for syncytiotrophoblast differentiation and placental development
Source: Cell Mol Life Sci. 2024 May 31;81(1):246. doi: 10.1007/s00018-024-05284-2 (PMC11143174; doi:10.1007/s00018-024-05284-2)
Supplement: Supplementary file 13 — Supplementary file13 (DOCX 3187 KB) [file 18_2024_5284_MOESM13_ESM.docx]

Supplemental information

**The GPI-anchor biosynthesis pathway is critical for syncytiotrophoblast differentiation and placental development**

Andrea Álvarez-Sánchez^1^[**^#^**](https://es.wikipedia.org/wiki/C_Sharp), Johanna Grinat^2^[**^#^**](https://es.wikipedia.org/wiki/C_Sharp), Paula Doria-Borrell^1^, Maravillas Mellado-López^1^, Érica Pedrera-Alcócer^1^, Marta Malenchini^1^, Salvador Meseguer^1^, Myriam Hemberger^3,4^, Vicente Pérez-García^1,5*^

**Affiliations:**

^1^ Centro de Investigación Príncipe Felipe, Eduardo Primo Yúfera, Valencia, Spain.

^2^ Epigenetics Programme, The Babraham Institute, Babraham Research Campus, Cambridge, United Kingdom.

^3^ Department of Biochemistry and Molecular Biology, Cumming School of Medicine, University of Calgary, Calgary, AB Canada.

^4^Alberta Children's Hospital Research Institute, University of Calgary, Calgary, Canada.

^5^Centro de Biología Molecular Severo Ochoa, CSIC-UAM, Madrid, Spain

[**#**](https://es.wikipedia.org/wiki/C_Sharp) These authors contributed equally to this work

***Corresponding author:**

Dr Vicente Perez-García

Centro de Investigación Príncipe Felipe

Calle de Eduardo Primo Yúfera, 3

46012 Valencia, Spain

[vperez@cipf.es](mailto:vperez@cipf.es)

List of material provided:

Supplemental Fig.S1: Analysis of placental defects in the *Pigl* and *Pigf* Knockout mice.

Supplemental Fig.S2: Molecular effects of *Pigl* and *Pigf* deficiency in mTSC.

Supplemental Fig.S3: The deletion of *Pigl* and *Pigf* impairs syncytiotrophoblast differentiation of mTSCs.

Supplemental Fig.S4: Validation of the GPI mutant gene signature in human placental samples from pregnancy disorder datasets.

**
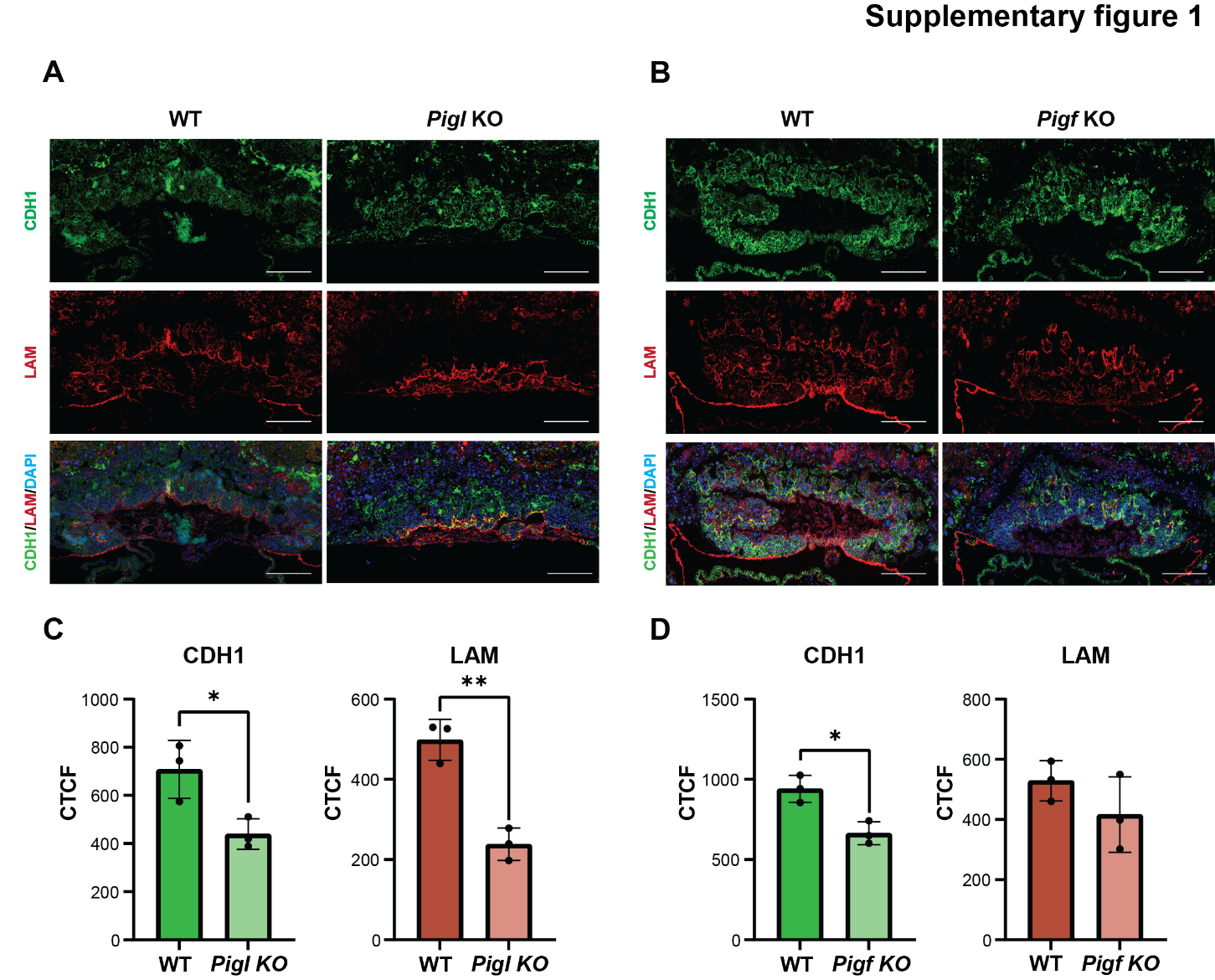
**

**Supplementary Figure 1:** **Analysis of placental defects in the *Pigl* and *Pigf* Knockout mice.**

Histological analysis of the placentas at E9.5 shows a strong placental defect in the syncytiotrophoblast layer development of *Pigl* KO mice **(A)** and *Pigf* KO mice **(B).** Immunofluorescence staining of corresponding placentas for E-Cadherin (CDH1) and basement membrane component LAMININ (Lam; demarcates fetal blood vessels). Nuclear counterstain with DAPI. Scale bar = 100um. Placental defects are representative of ≥3 independent mutants per genotype. **C, D)** The bar plots show the mean quantification of CDH1 and LAMININ staining for *Pigl* KO (C) and *Pigf* KO (D) placentas compared to littermate wildtype (WT) controls, measured as CTCF (Corrected total cell fluorescence). Individual data points are shown as dots and error bars show standard deviation. Pairwise comparisons were done by a two-tailed t-test (*p<0.05, **p<0.01).

**
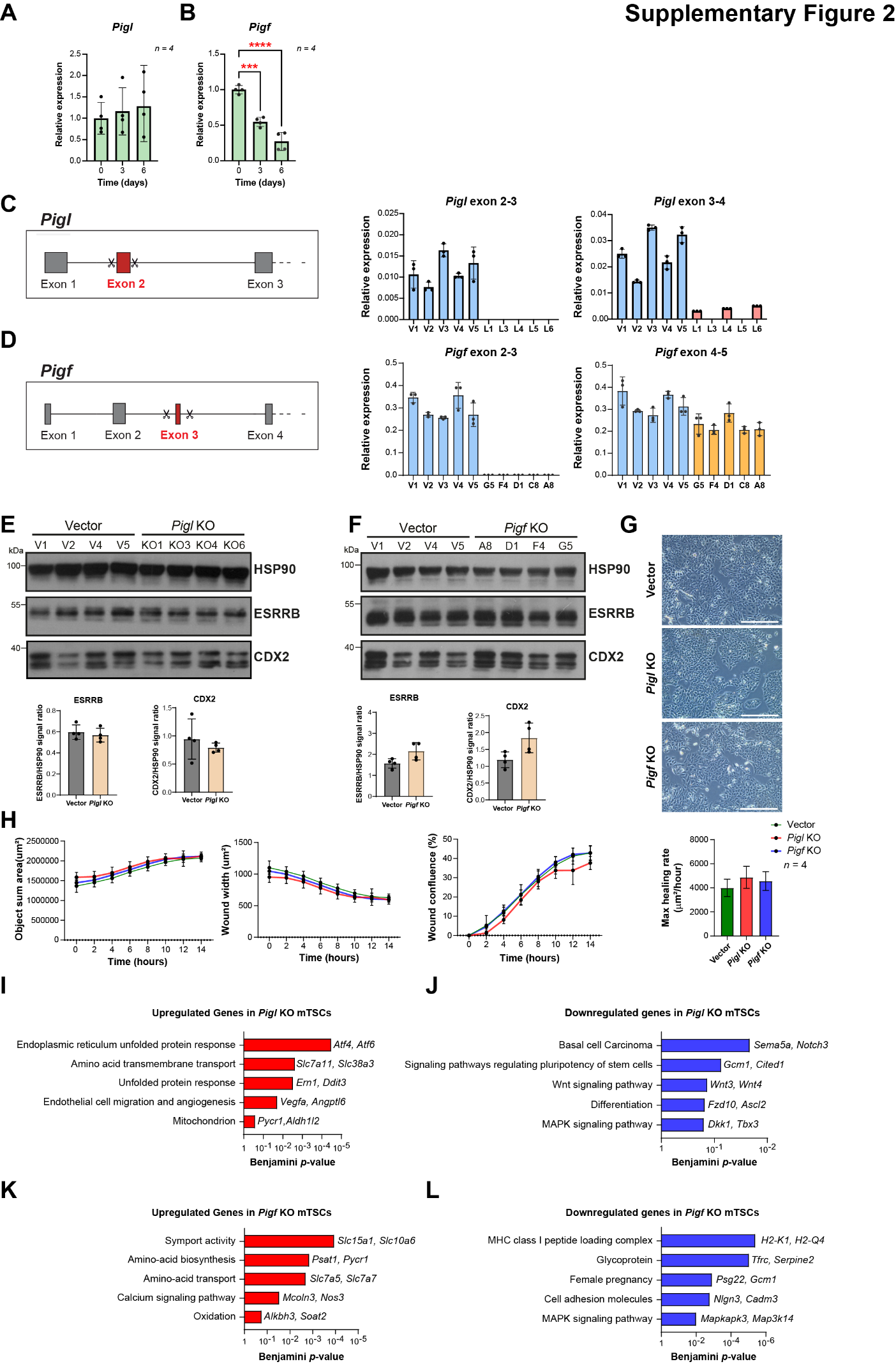
**

**Supplementary figure 2: Molecular effects of *Pigl* and *Pigf* deficiency in mTSC**. **A, B)** RT-qPCR analysis of *Pigl* **(A)** and *Pigf* **(B)** expression during a 6-day differentiation time course of mouse trophoblast stem cells (mTSCs). Data are normalized to *Sdha* and are displayed as mean of four replicates ± SEM; ***p<0.001 (one-way ANOVA with Dunnett’s multiple comparisons test). **C, D)** Details of the CRISPR/Cas9 KO strategy for deleting *Pigl* **(C)** and *Pigf*  **(D)** genes. RT–qPCR analyses were performed on single-cell expanded mTSC clones to confirm homozygous Knockout (KO). Data are mean ± SEM of n = 3 technical replicates. **E, F)** Western blot analysis of the trophoblast stem cell markers CDX2 and ESRRB protein levels in vector control and *Pigl^-/-^* **(E)** and *Pigf^-/-^* **(F)** mTSCs grown in stem cell conditions. HSP90 was used as loading control. The graphs show the quantification of four independent biological (cell clones) replicates. Data are mean ± SEM; *p<0.05 (Student’s t-test). **G)** Morphology analysis of Vector and *Pigl*- and *Pigf*-null mTSCs growing in stem cell conditions. No changes in epithelial morphology were observed. Scale bar: 100 µm. **H)** Wound healing assay on *Pigl*- and *Pigf*-null mTSCs growing in stem cell conditions. The graphs show the quantification of the kinetic cell area coverage values (Object Sum Area) that were used to generate three additional wound healing metrics including wound width, wound confluence, and maximum wound healing rate. Data are mean ± SEM of n = 4 independent biological (cell clones) replicates. **I, J)** Gene ontology analyses of upregulated **(I)** and downregulated genes **(J)** in *Pigl* knockout (KO) compared to vector control mTSCs, identified by DESeq2 and >1-log2 Fold change in expression. **K, L)** Equivalent analysis for *Pigf* KO mTSCs**.**

**
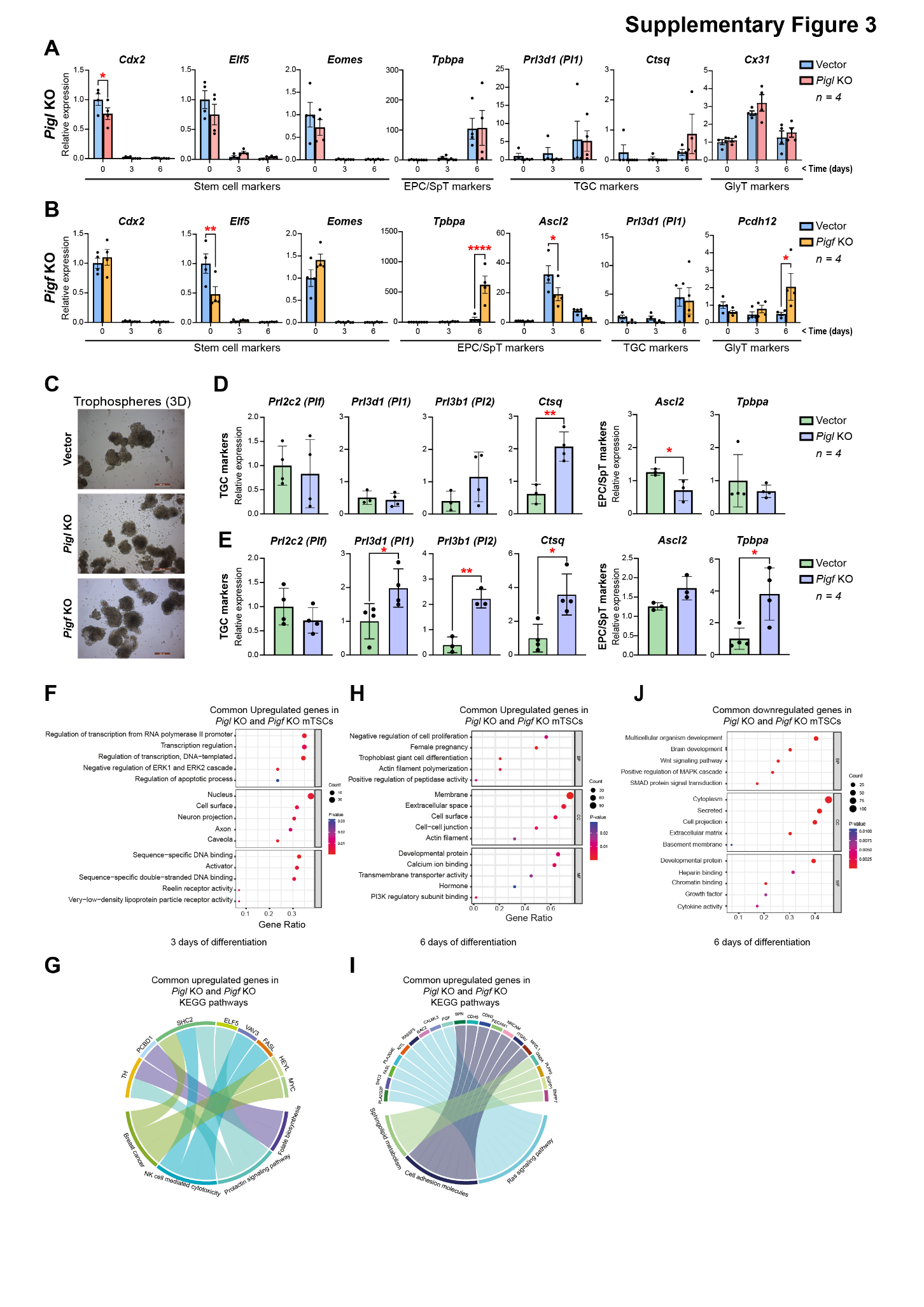
**

**Supplementary figure 3: The deletion of *Pigl* and *Pigf* impairs syncytiotrophoblast differentiation of mTSCs. A)** Analysis of Vector and *Pigl*^-/-^ and *Pigf*^-/-^ mTSCs grown in self-renewal conditions (0 days) or after differentiation for 3 and 6 days assessed by RT-qPCR. Data are mean ± SEM of *n =* 4 independent biological (cell clones) replicates. **B)** Equivalent analysis for *Pigf*^-/-^ mTSCs. *p<0.05, **p<0.01, ***p<0.001, ****p<0.0001 (two-way ANOVA with Sidak’s multiple comparisons test). **C)** Morphology of 3D-trophospheres after 8 days of differentiation in low attachment conditions. Representative images of 3 independent vector control and *Pigl* and *Pigf* KO cell clones. Scale bar: 200 µm. **D)** RT-qPCR analysis of 3D-trophospheres generated from vector control and*Pigl* and Pigf-null mTSCs after 8 days of differentiation. **E)** Equivalent analysis for *Pigf*^-/-^ mTSCs Data are normalized to *Sdha* and are displayed as the mean of *n =* 4 independent biological (cell clones) replicates ± SEM; *p<0.05, **p<0.01 (Student’s t-test). **F)** GO enrichment analysis of the commonly upregulated genes in *Pigl* KO and *Pigf* KO mTSCs after 3 days of differentiation. **G)** Chord plot indicates the relationship between genes and KEGG pathways in the commonly upregulated genes in *Pigl* KO and *Pigf* KO mTSCs after 3 days of differentiation. **H, I)** Equivalent analysis for the commonly upregulated genes in *Pigl* KO and *Pigf* KO mTSCs after 6 days of differentiation. **J)** GO enrichment analysis of the commonly downregulated genes in *Pigl* KO and *Pigf* KO mTSCs after 6 days of differentiation.

**
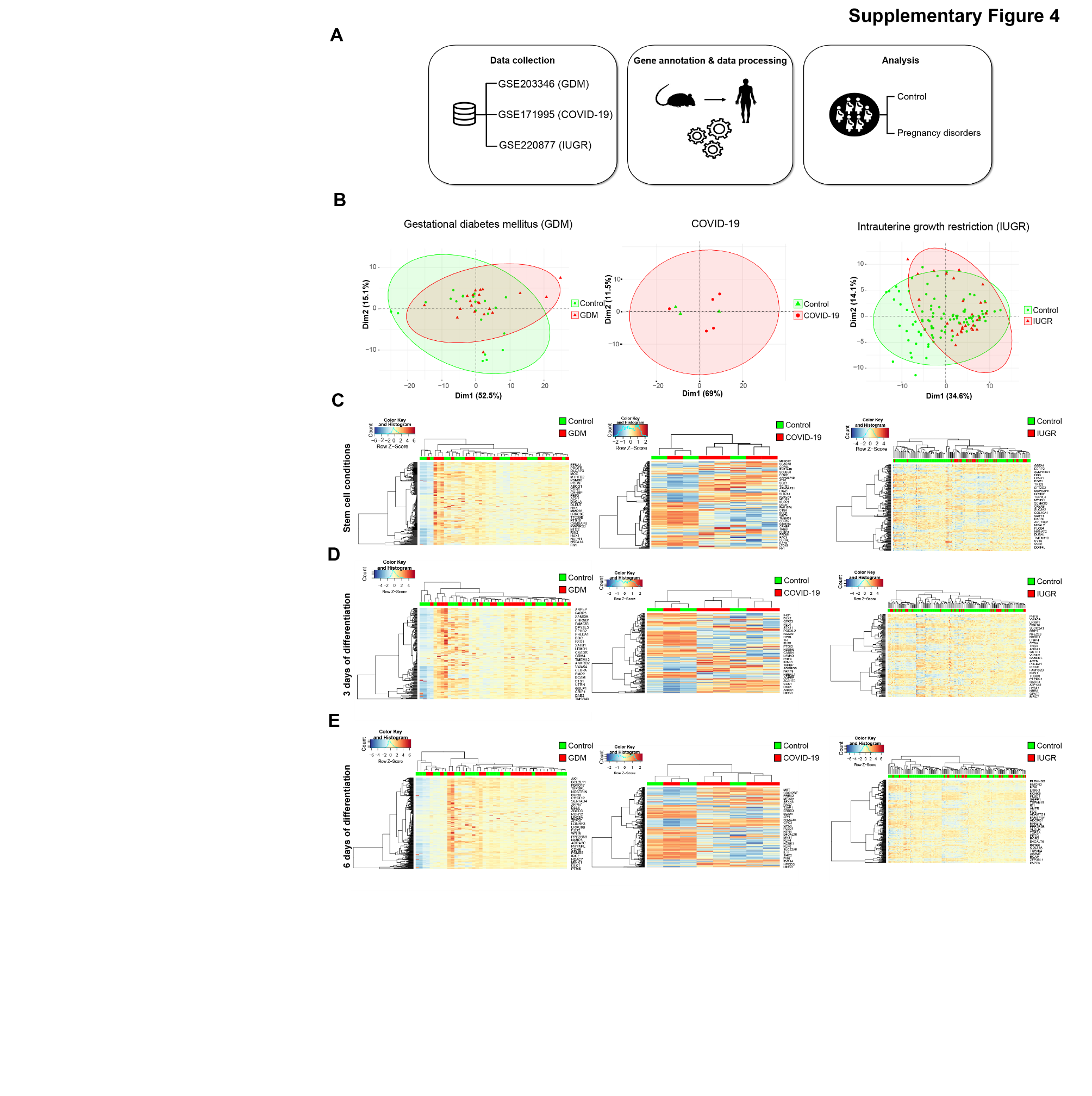
Supplementary Figure 4:** **Validation of the GPI mutant gene signatures in human placental samples from pregnancy disorder datasets.** **A)** Schematic diagram of the study design. RNAseq data from Gestational Diabetes Mellitus (GDM) (GSE203346), COVID-19 (GSE171995), and Intrauterine Growth Restriction (IUGR) (GSE220877) datasets were collected from GEO database. Gene annotation and data processing including the normalization of RNA-seq counts matrix was applied in order to analyze the footprint signature. Principal component analysis (PCA) and unsupervised clustering validated the potential of the list of genes to separate the groups in each study. **B)** PCA plots for the GDM, COVID-19, and IUGR (red) and control (green) samples of the three datasets, based on the GPI gene signature of mTSCs grown in stem cell conditions. X and Y axis show dimension 1 and dimension 2 that explain the % of the total variance. **C**) Unsupervised clustering based on the GPI gene signature of mTSCs grown in stem cell conditions across GDM, COVID-19 and IUGR (red) and control (green) samples of the three datasets **D**) Equivalent analysis for the commonly deregulated genes in *Pigl* KO and *Pigf* KO mTSCs after 3 days of differentiation. **E**) Equivalent analysis for the commonly deregulated genes in *Pigl* KO and *Pigf* KO mTSCs after 6 days of differentiation. GDM, Gestational Diabetes Mellitus; IUGR, Intrauterine Growth Restriction.
